# A family of partial-linear single-index models for analyzing complex environmental exposures with continuous, categorical, time-to-event, and longitudinal health outcomes

**DOI:** 10.1186/s12940-020-00644-4

**Published:** 2020-09-11

**Authors:** Yuyan Wang, Yinxiang Wu, Melanie H. Jacobson, Myeonggyun Lee, Peng Jin, Leonardo Trasande, Mengling Liu

**Affiliations:** 1 Department of Population Health, NYU Langone Health, 180 Madison Avenue, New York, NY 10016 USA; 2 Department of Pediatrics, NYU Langone Health, New York, NY USA; 3 Department of Environmental Medicine, NYU Langone Health, New York, NY USA

**Keywords:** Environmental mixtures, NHANES, Semiparametric model, Triglyceride

## Abstract

**Background:**

Statistical methods to study the joint effects of environmental factors are of great importance to understand the impact of correlated exposures that may act synergistically or antagonistically on health outcomes. This study proposes a family of statistical models under a unified partial-linear single-index (PLSI) modeling framework, to assess the joint effects of environmental factors for continuous, categorical, time-to-event, and longitudinal outcomes. All PLSI models consist of a linear combination of exposures into a single index for practical interpretability of relative direction and importance, and a nonparametric link function for modeling flexibility.

**Methods:**

We presented PLSI linear regression and PLSI quantile regression for continuous outcome, PLSI generalized linear regression for categorical outcome, PLSI proportional hazards model for time-to-event outcome, and PLSI mixed-effects model for longitudinal outcome. These models were demonstrated using a dataset of 800 subjects from NHANES 2003–2004 survey including 8 environmental factors. Serum triglyceride concentration was analyzed as a continuous outcome and then dichotomized as a binary outcome. Simulations were conducted to demonstrate the PLSI proportional hazards model and PLSI mixed-effects model. The performance of PLSI models was compared with their counterpart parametric models.

**Results:**

PLSI linear, quantile, and logistic regressions showed similar results that the 8 environmental factors had both positive and negative associations with triglycerides, with a-Tocopherol having the most positive and trans-b-carotene having the most negative association. For the time-to-event and longitudinal settings, simulations showed that PLSI models could correctly identify directions and relative importance for the 8 environmental factors. Compared with parametric models, PLSI models got similar results when the link function was close to linear, but clearly outperformed in simulations with nonlinear effects.

**Conclusions:**

We presented a unified family of PLSI models to assess the joint effects of exposures on four commonly-used types of outcomes in environmental research, and demonstrated their modeling flexibility and effectiveness, especially for studying environmental factors with mixed directional effects and/or nonlinear effects. Our study has expanded the analytical toolbox for investigating the complex effects of environmental factors. A practical contribution also included a coherent algorithm for all proposed PLSI models with R codes available.

## Background

Humans are constantly exposed to a mixture of environmental factors that have the potential to affect health adversely or beneficially, such as chemical contaminants, air pollutants, dietary factors, and behavioral and socioeconomic characteristics. The *exposome*, which is defined as the totality of environmental (non-genetic) exposures from conception onwards (i.e., environmental factors), has been proposed to address the complexities related to studying multiple exposures [1]. It is well acknowledged that single-exposure-outcome approaches do not allow for the disentangling of effects of multiple exposures, and miss the interplay among them [2]. Therefore, quantifying the complex effects of multiple and simultaneous environmental exposures on health outcomes has become a focus of environmental health research [3, 4]. The National Institute of Environmental Health Sciences (NIEHS) has been supporting and conducting combined exposure research, and highlighted this direction as a priority in its 2018–2023 Strategic Plan [5].

Statistical approaches have been proposed to assess the effects of multiple exposures on health outcomes from different perspectives, each focusing on distinct scientific questions [2, 6]. However, several challenges for statistical modeling are apparent in these investigations [2]. First, multiple environmental exposures occur simultaneously, often with complex correlation structures among them. Second, they may exhibit synergistic or antagonistic effects on the health outcome, and their associations with health outcomes can be positive, negative, or null, which reflect the complex web of physiological relationships and/or “reverse causality” [7, 8]. Third, the relationships between environmental factors and health outcomes can be non-linear, which pose challenges to standard parametric regression-based methods [9]. Fourth, it is well recognized that statistical methods have different strengths in addressing various aspects of scientific investigations. For example, from the methodology perspective, Stafoggia et al. [2] classified the statistical methods for analysis of environmental mixtures into dimension reduction, variable selection, or grouping or clustering. From the view of scientific questions, Gibson et al. [4] distinguished different study objectives as: identifying the important components in the mixtures, studying synergistic effects, or characterizing the overall effect of the mixtures.

Specifically, in studying the joint effects of environmental exposures, weighted quantile sum regression (WQS) [9, 10] and Bayesian kernel machine regression (BKMR) [11, 12] are two popular modeling approaches. The WQS method is a parametric method assuming that all exposures are associated with the outcome in one direction in each run of analysis, and then derives a one-dimensional weighted sum score of the exposures under the assumed direction for the estimation of overall effect. BKMR is a nonparametric method and can handle nonlinear and complex relationships between exposure mixtures and outcome. Some measures have been proposed to quantify the importance and effects of exposure components based on BKMR results. For example, the posterior inclusion probability (PIP) characterizes the probability of an exposure being associated with outcome, and change per interquartile range increase quantifies the expected change in the outcome in association with the change in an exposure from the 25th to 75th percentile, while other exposures are fixed to the median. However, the nonparametric exposure-response function may be difficult to interpret and its fitting often needs a large sample size [13, 14]. In addition, WQS and BKMR have been generalized to study environmental mixtures with several types of outcomes, such as WQS for longitudinal outcomes [15] and BKMR for time-to-event outcomes [16]. However, a general modeling framework that can alleviate the above limitations in environmental health research is still desired [17].

Partial-linear single-index (PLSI) models are a family of semiparametric models that reside between the completely unstructured nonparametric models and restrictive parametric regression models [18–20]. By reducing multiple exposures into a single index through a linear combination of the exposures, the PLSI models can reduce the “curse of dimensionality” issue and improve modeling efficiency. The application and performance of single-index linear regression for analysis of environmental exposures with continuous outcomes has been evaluated previously (pending publication). Specifically, the PLSI modeling framework allows the associations between exposures and outcomes to be in the positive or negative direction, provides explicit and interpretable quantification on the relative direction and importance of the exposures, and models these effects with flexibility through a nonparametric link function. Therefore, PLSI models are able to address the objectives of identifying important individual exposures, their direction and magnitude of association with the outcome, and characterizing the overall effect of multiple exposures or exposure mixtures, responding well to the key scientific objectives summarized by Gibson et al. [4]. In recent years, research on PLSI models has attracted increasing attention and extended to different types of outcomes, such as categorical [21–23], time-to-event [24–27] and longitudinal [28–31] outcomes. Table 1 summarizes the outcome types of interest and corresponding PLSI models with key references and their corresponding counterpart parametric models.

**Table 1:** Summary of outcome types and corresponding PLSI models and parametric models

Outcome type	PLSI models	Counterpart models	Key references	Equation
Continuous	PLSI linear regression	Linear regression	[18, 21, 22, 32–38]	(1)
	PLSI quantile regression	Quantile regression	[39–44]	(2)
Categorical (binary)	PLSI generalized linear (logistic) regression	Generalized linear (logistic) regression	[18, 22, 36, 38]	(3)
Time-to-event	PLSI PH model	Cox PH model	[24–27]	(4)
Longitudinal	PLSI mixed-effects model	Linear mixed-effects model	[28, 29, 45–47]	(5)

The main goal of this study was to unify the resource advantages of PLSI models into one general framework for analyzing environmental factors, and to demonstrate their values in environmental research for different types of health outcomes. We exemplified the use of PLSI models in assessing the associations between correlated environmental factors with health outcomes using real and simulated datasets based on National Health and Nutrition Examination Survey (NHANES) 2003–2004 cycle. Another aim was to develop effective computation algorithms for the PLSI models and to consolidate these models using R packages.

## Methods

### NHANES dataset

**Fig. 1:**
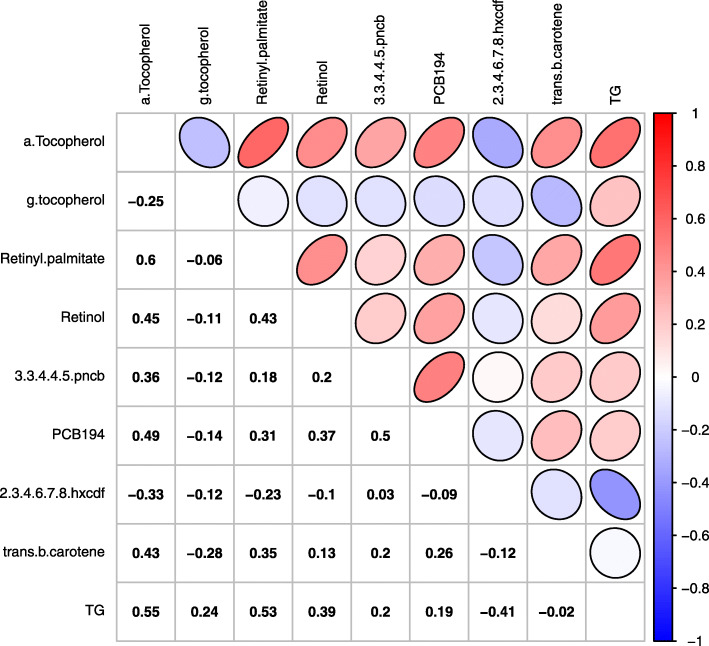
Correlation matrix of Pearson correlation coefficients of 8 factors and triglycerides in NHANES 2002–2003 (*N* = 800)

To demonstrate the PLSI models, we used the data from the NHANES 2003–2004 cycle based on the original paper by Patel et al. [48], which systematically evaluated the associations of environmental factors with serum lipid levels. We used serum triglyceride concentrations as the primary outcome for demonstration and also considered three demographic variables, age, sex, and race/ethnicity as potential confounders. Participants with data on serum triglycerides, environmental factors and confounders were included in this study (*n* = 800). Details on data pre-processing are provided in Additional file 1: Figure S1. Subjects provided written informed consent, and the Institutional Review Board of the National Center for Health Statistics approved the survey [49]. Table 2 summarizes the final variables included in analyses, and Fig. 1 shows the correlation matrix of the final 8 environmental factors and triglycerides. The dataset is provided as Additional file 2, and the R codes conducting data cleaning is included in the R markdown file (Additional file 3).

**Table 2:** List of analyzed variables from NHANES 2002–2003 dataset

Type	Variable name	Abbreviations	Symbol
Outcome			
	Triglycerides (mg/dL)	TG	Y
Environmental factors			
	a-Tocopherol (ug/dL)	a-Tocopherol	X1
	g-tocopherol (ug/dL)	g-tocopherol	X2
	Retinyl palmitate (ug/dL)	Retinyl-palmitate	X3
	Retinol (ug/dL)	Retinol	X4
	3,3′,4,4′,5-Pentachlorobiphenyl (pncb) Lipid Adj (pg/g)	3,3,4,4,5-pncb	X5
	Polychlorinated Biphenyl (PCB) 194 Lipid Adj (ng/g)	PCB156	X6
	2,3,4,6,7,8-hxcdf Lipid Adj (pg/g)	2,3,4,6,7,8-hxcdf	X7
	trans-b-carotene (ug/dL)	trans-b-carotene	X8
Confounders			
	Age (years)	Age	Z1
	Sex (1: male; 2: female)	Sex	Z2
	Race/Ethnicity (1: Non-Hispanic white; 2: Non-Hispanic black; 3: Mexican American; 4: Other race - Including multi-racial; 5: Other Hispanic)	Race	Z3

### Notation and PLSI models overview

For notational convention throughout this article, we let *Y* denote the outcome, *X* = (*X*_1_, …, *X*_8_) denote the 8 exposure variables to be modeled into the “single index” term, and vector *Z* represent the confounders (age, sex, and race/ethnicity). The outcome, continuous triglycerides, and all exposure variables, except for retinol, were log-transformed, and all exposure variables were standardized to have mean of zero and standard deviations of 1 before model fitting.

In contrast to standard generalized linear models (GLMs) that specify the effects of exposures and confounders all linearly as *β*^′^*X* + *γ*′*Z*, PLSI models assume the influence of exposures *X* through a nonparametric link function on the single index while modeling other confounders linearly, i.e. *g*(*β*^′^*X*) + *γ*′*Z*. The single index coefficients *β*^′^ s characterize the relative direction and importance of each exposure *X*_*i*_, and γ for the corresponding linear coefficient vector for confounder vector *Z*. Because the link function *g*(·) is completely nonparametric, to ensure model identifiability, the *l*_2_ norm of *β*′s (i.e. $\sqrt{\beta_1^2+\dots +{\beta}_8^2}$) is set to be 1 with the first component *β*_1_ > 0, which are the commonly used parametrization constraints for all PLSI models [22, 36–38]. PLSI models are not identifiable without these constraints because any scaling or constant shift can be absorbed by the nonparametric link function.

### Continuous outcome: mean regression

The PLSI linear regression model is considered as a generalization of both standard linear regression and missing-link function problem in linear modeling [50], and specified as

1 $$ Y=g\left(\sum \limits_{j=1}^8{\beta}_j{X}_j\right)+{\upgamma}^{\prime}\mathrm{Z}+\varepsilon $$

The semiparametric PLSI linear regression has the parametric component ${\sum}_{j=1}^8{\beta}_j{X}_j$ and γ^′^Z for easy linear representation and interpretation, and the nonparametric components *g*(∙) is totally unspecified and represents the overall effect of single index, which incorporates potential nonlinearity and interactions among exposures. When the estimated *g*(∙) is monotone, the effect of *X*_*j*_ can be interpreted qualitatively using the sign of *β*_*j*_. If *g*(∙) is monotone increasing, then a positive sign for *β*_*j*_ suggests increased conditional expectation of *Y* at larger value of *X*_*j*_, and vice versa for a negative sign. As the overall scale of *β* is set, ∣*β*_*j*_∣ can be explained as the relative importance of *X*_*j*_ affecting the mean of outcome *Y* as *X*_*j*_ is perturbed while *g*(∙) and other variables are held fixed. We can also intuitively interpret ${\beta}_j^2$ as the proportion of contribution to the single index by variable *X*_*j*_ because, when (*X*_1_, *X*_2_, …, *X*_8_) are independent, ${\beta}_j^2$ simply represents *X*_*j*_ ’s variance contribution.

Besides the analysis for the 8 selected exposures, we also conducted a sensitivity analysis including all 22 environmental factors to investigate the performance of PLSI linear regression to handle highly correlated exposures (Additional file 1: Figure S2).

### Continuous outcome: quantile regression

Beyond the commonly-considered effects of environmental factors on the mean of a continuous outcome, sometimes we are interested in the specific relations cross multiple points of the outcome’s distribution, such as higher quantiles of triglycerides [51], higher quantiles of blood pressure [52], low quantiles of birth weight [53], or lower quantiles of intelligence quotient scores [54]. Moreover, when the distribution of continuous outcome deviates from Gaussian, modeling the median can be more robust than evaluating the mean by conventional linear regression [55]. For this purpose, quantile regression (QR), which was originally proposed by Koenker and Bassett [56] and used as a useful technique in econometrics [57] and growth curve analysis [58], enables us to study the associations of environmental factors with continuous health outcomes as various quantiles across its distribution. PLSI quantile regression is a combination of the PLSI technique and QR [42, 43], and thus we consider it for the analysis of joint effects of multiple environmental factors on the quantile(s) of continuous outcome variable.

Given a specific *τ* ∈ (0, 1), the PLSI quantile regression for the τth conditional quantile *θ*_*τ*_ of continuous outcome *Y* given environmental factors *X* and covariates *Z* can be specified as

2 $$ {\theta}_{\tau}\left(Y|X,Z\right)={\mathrm{g}}_{\tau}\left(\sum \limits_{j=1}^8{\beta}_{\tau j}{X}_j\right)+{\gamma}_{\tau}^{\prime }Z $$

Interpretation of coefficients *β*_*τ*_ ′ s in the PLSI quantile regression is similar to that of PLSI linear regression, with the difference being that the associations are now with the conditional quantiles of outcome variable *θ*_*τ*_(*Y*| *X*, *Z*) instead of the mean.

### Categorical outcome: generalized linear regression

PLSI generalized linear regression can be employed for categorical outcomes, such as binary, multinomial, or count variables. Here we considered the binary outcome of high triglycerides (> 150 mg per deciliter) [59], which accounted for 30.75% of the 800 subjects. The PLSI logistic model is specified as

3 $$ logit(P(Y=1|X,Z)) = g \left(\sum_{j=1}^{8}\beta_{j}X_{j}\right) + {\gamma}^{\prime}\mathrm{Z} $$

The interpretation of coefficients is based on the log odds that response value is ‘1’ conditioning on the predictors, and *β*_*j*_ represents the relative direction and importance of *X*_*j*_ associated with the log odds of high triglycerides when scale of *β* is set and *g*(∙) and other variables are held fixed. The logit function can be adapted accordingly to the type of categorical outcome, and the model specifications for multinomial and count outcomes were provided in Additional file 1: Table S1.

### Time-to-event outcome: proportional hazards model

The Cox proportional hazards (PH) regression has been the pivotal model in time-to-event analysis since Sir Cox proposed it in 1972 [60, 61]. The Cox PH regression models the hazard function and assumes that covariates have linear effects on the log hazard function. Combining PLSI modeling technique and Cox PH regression, the PLSI PH model is specified as

4 $$ \lambda \left(t\left|X,Z\right.\right)={\lambda}_0(t)\exp \left\{g\left(\sum \limits_{j=1}^8{\beta}_j{X}_j\right)+{\upgamma}^{\prime}\mathrm{Z}\right\}, $$

where *β*_*j*_ can be explained as the relative effect direction and importance of *X*_*j*_ on the log hazard function and *g*(∙) characterizes the overall effect of the index.

### Longitudinal outcome: mixed-effects model

Longitudinal studies arise frequently in environmental research, in which outcomes are measured repeatedly over a period of time with either baseline or time-dependent environmental factors. As measurements from the same subject are often correlated, subject-specific random effects are used to accommodate within-subject dependence and to explain across-subject heterogeneity. Mixed-effects models provide a general and flexible framework for modeling longitudinal data, consisting of two modeling components: fixed effects and random effects, characterizing the population mean and individual variation, respectively [62, 63]. Mixed-effects models in general are amenable to missing data and can accommodate missing completely at random or missing at random [62, 64]. Without loss of generality, we consider a longitudinal study with *N* subjects and the *i*th subject has *n*_*i*_ observations over time. Repeated measures of the outcome are denoted by *Y*_*ij*_, exposure vector *X*_*ij*_, covariate vector *Z*_*ij*_ and observation time *T*_*ij*_, and then the observed full dataset is {(*Y*_*ij*_, *X*_*ij*_, *Z*_*ij*_, *T*_*ij*_), *i* = 1, …, *N*, *j* = 1, …, *n*_*i*_}.

Specifically, the PLSI mixed-effects model with a random intercept is specified as

5 $$ {Y}_{i\;j}=g\left(\sum \limits_{l=1}^8{\beta}_l{X}_{i\;j\;l}\right)+{Z}_{i\;j}^{\prime}\upgamma +{b}_i+\omega {T}_{i\;j}+{\varepsilon}_{i\;j}, $$

where *b*_*i*_ represents the subject-specific random intercept and *ω* represents the time effect on the outcome. Note that PLSI mixed-effects model can accommodate additional random effects and other model specifications of fixed effects and interactions, and the model specification for a PLSI mixed-effects model with a random slope was provided in Additional file 1: Table S1. The index coefficient *β*_*l*_ can be explained as the relative direction and importance of *X*_*ijl*_ as *X*_*ijl*_ is perturbed when scale of *β* is set and *g*(∙) and other variables are held fixed, and *g*(∙) represents the overall effect of the single index with the mean of longitudinal outcome.

### Simulation settings

Since the NHANES survey dataset does not have time-to-event outcome nor longitudinal outcome, we conducted simulations to demonstrate the PLSI PH model and PLSI mixed-effects model. The coefficients for the 8 environmental factors and three confounding variables were set based on the results from the PLSI linear regression for continuous triglycerides. We kept the original direction of these associations and the absolute rank for each environment factor, and set the effect sizes in a wider range to be more distinguishable (see details in Tables 3 and 4). Moreover, we considered the link function *g*(.) to be either *g*(*x*) = *x* to facilitate the direct comparison with the parametric models, or as a quadratic function *g*(*x*) = *x*^2^ to mimic the scenario with nonlinear effects and pair-wise interactions between the exposures as $g\left({\sum}_{j=1}^8{\beta}_j{X}_j\right)={\beta}_1^2{X}_1^2+\dots +{\beta}_8^2{X}_8^2+2{\beta}_1{\beta}_2{X}_1{X}_2+\dots +2{\beta}_7{\beta}_8{X}_7{X}_8$, or a more complex function *g*(*x*) = 0.2*x*^3^ − *x*^2^ + 3*x* to demonstrate higher-order nonlinear effects and interactions, such as three-way interactions. Furthermore, we visualized the interaction effects of two variables by plotting the stratified effect of one variable when fixing the other variables at various levels. Time-to-event outcomes were generated using model (4) with *λ*_0_ = 1 in the identity link function scenario, *λ*_0_ = 1/ exp(2) in the quadratic link function scenario and in the cubic polynomial link function scenario; with a censoring rate as 20% in all of them. Longitudinal outcomes were generated using model (5) with *t*_*ij*_ ranged [1, 6] and *ω* = 1. The number of possible observations for each subject was assumed to vary randomly between 2 and 6. The errors followed a first order autoregressive process (i.e. AR(1)), with the autocorrelation as 0.4 and standard deviation as 1.5 to mimic decreasing dependence with time. All details of data generation used in these simulations are included in the R markdown file (Additional file 3).

**Table 3:** Simulation results from PLSI PH model and Cox PH model

Variable	True rank	True coefficient	PLSI PH rank	PLSI PH estimate	PLSI PH 95% CI	PLSI PH Proportion of contribution (%)	Cox PH rank	Cox PH original estimate	Cox PH original 95% CI	Cox PH normed estimate	Cox PH normed 95% CI
Identity link function											
Environmental factors											
a-Tocopherol	1	0.560	1	0.546	(0.437, 0.656)	29.9	1	0.558	(0.428, 0.688)	0.546	(0.446, 0.646)
g-tocopherol	2	0.490	2	0.500	(0.427, 0.572)	25.0	2	0.511	(0.417, 0.605)	0.500	(0.428, 0.571)
Retinyl-palmitate	3	0.420	3	0.408	(0.297, 0.520)	16.7	3	0.418	(0.310, 0.526)	0.409	(0.301, 0.516)
Retinol	7	0.140	7	0.122	(0.029, 0.216)	1.5	7	0.125	(0.029, 0.221)	0.122	(0.034, 0.210)
3,3,4,4,5-pncb	8	0.070	8	0.059	(−0.039, 0.158)	0.4	8	0.061	(− 0.040, 0.161)	0.059	(− 0.033, 0.151)
PCB194	6	−0.210	6	−0.207	(− 0.346, − 0.068)	4.3	6	− 0.212	(− 0.351, − 0.074)	− 0.208	(− 0.329, − 0.087)
2.3.4.6.7.8.hxcdf	5	− 0.280	5	− 0.270	(− 0.356, − 0.183)	7.3	5	− 0.275	(− 0.367, − 0.183)	− 0.269	(− 0.354, − 0.185)
trans.b.carotene	4	− 0.350	4	− 0.388	(− 0.467, − 0.310)	15.1	4	− 0.397	(− 0.493, − 0.302)	−0.389	(− 0.465, − 0.313)
Covariates											
Age		0.005		0.009	(0.001, 0.017)			0.009	(0.002, 0.016)		
Sex (female)		−0.076		−0.039	(− 0.216, 0.138)			−0.039	(− 0.217, 0.138)		
Race/Ethnicity											
Non-Hispanic white		Ref		Ref				Ref			
Non-Hispanic black		−0.138		−0.135	(− 0.367, 0.097)			−0.135	(− 0.361, 0.091)		
Mexican American		0.175		0.114	(−0.116, 0.344)			0.114	(−0.107, 0.335)		
Other race		0.409		0.528	(0.118, 0.937)			0.528	(0.077, 0.978)		
Other Hispanic		0.355		0.477	(−0.021, 0.975)			0.477	(0.018, 0.936)		
Quadratic link function											
Environmental factors											
a-Tocopherol	1	0.560	1	0.526	(0.403, 0.648)	27.6	1	0.289	(0.124, 0.455)	0.861	(0.621, 1.101)
g-tocopherol	2	0.490	2	0.513	(0.296, 0.730)	26.3	3	0.098	(−0.011, 0.207)	0.292	(−0.024, 0.607)
Retinyl-palmitate	3	0.420	3	0.445	(0.231, 0.659)	19.8	6	0.037	(−0.088, 0.161)	0.109	(−0.253, 0.470)
Retinol	7	0.140	7	0.161	(0.041, 0.281)	2.6	4	−0.041	(− 0.154, 0.072)	− 0.122	(− 0.465, 0.222)
3,3,4,4,5-pncb	8	0.070	8	0.061	(−0.023, 0.146)	0.4	8	0.013	(−0.102, 0.128)	0.040	(− 0.305, 0.384)
PCB194	6	−0.210	6	−0.208	(−0.322, − 0.093)	4.3	7	0.020	(−0.132, 0.172)	0.059	(− 0.338, 0.457)
2.3.4.6.7.8.hxcdf	5	− 0.280	5	−0.252	(− 0.392, − 0.113)	6.4	5	− 0.039	(− 0.138, 0.061)	− 0.115	(− 0.445, 0.215)
trans.b.carotene	4	− 0.350	4	− 0.355	(− 0.477, − 0.234)	12.6	2	− 0.120	(− 0.228, − 0.012)	−0.358	(− 0.637, − 0.079)
Covariates											
Age		0.005		0.003	(−0.002, 0.008)			−0.005	(−0.012, 0.003)		
Sex (female)		− 0.076		−0.081	(−0.269, 0.108)			−0.103	(−0.297, 0.092)		
Ethnicity											
Non-Hispanic white		Ref		Ref				Ref			
Non-Hispanic black		−0.138		0.044	(− 0.211, 0.299)			0.083	(−0.154, 0.320)		
Mexican American		0.175		0.100	(−0.152, 0.352)			0.125	(−0.118, 0.369)		
Other race		0.409		0.186	(−0.438, 0.811)			−0.189	(−0.722, 0.345)		
Other Hispanic		0.355		0.096	(−0.567, 0.759)			−0.096	(− 0.634, 0.442)

**Table 4:** Simulation results from PLSI mixed-effects model and linear mixed-effects model

Variable	True rank	True coefficient	PLSI ME rank	PLSI ME estimate	PLSI ME 95% CI	PLSI ME Proportion of contribution (%)	Linear ME rank	Linear ME original estimate	Linear ME original 95% CI	Linear ME normed estimate	Linear ME normed 95% CI
Identity link function											
Environmental factors											
a-Tocopherol	1	0.560	1	0.584	(0.469, 0.698)	34.1	1	0.590	(0.456, 0.723)	0.580	(0.519, 0.642)
g-tocopherol	2	0.490	2	0.481	(0.396, 0.566)	23.1	2	0.490	(0.401, 0.579)	0.482	(0.439, 0.525)
Retinyl-palmitate	3	0.420	3	0.402	(0.284, 0.520)	16.2	3	0.408	(0.302, 0.513)	0.401	(0.336, 0.467)
Retinol	7	0.140	7	0.091	(−0.025, 0.206)	0.8	7	0.088	(−0.011, 0.186)	0.086	(0.027, 0.145)
3,3,4,4,5-pncb	8	0.070	8	0.054	(−0.067, 0.175)	0.3	8	0.058	(−0.047, 0.164)	0.057	(0.000, 0.114)
PCB194	6	−0.210	6	−0.225	(−0.378, − 0.072)	5.1	6	− 0.236	(− 0.372, − 0.099)	−0.232	(− 0.303, − 0.160)
2.3.4.6.7.8.hxcdf	5	− 0.280	5	− 0.236	(− 0.344, − 0.128)	5.6	5	−0.241	(− 0.331, − 0.151)	−0.237	(− 0.295, − 0.179)
trans.b.carotene	4	− 0.350	4	− 0.386	(− 0.475, − 0.297)	14.9	4	−0.392	(− 0.486, − 0.298)	−0.386	(− 0.433, − 0.339)
Covariates											
Intercept		0.000		−0.069	(−0.426, 0.287)			−0.074	(−0.486, 0.339)		
Age		0.005		0.011	(0.004, 0.019)			0.011	(0.005, 0.018)		
Sex (female)		−0.076		−0.121	(− 0.245, 0.003)			−0.125	(− 0.302, 0.051)		
Race/Ethnicity											
Non-Hispanic white		Ref		Ref				Ref			
Non-Hispanic black		−0.138		−0.225	(−0.368, − 0.082)			− 0.231	(− 0.450, − 0.012)		
Mexican American		0.175		0.030	(−0.123, 0.184)			0.027	(−0.195, 0.249)		
Other race		0.409		0.086	(−0.231, 0.403)			0.081	(−0.395, 0.557)		
Other Hispanic		0.355		0.811	(0.463, 1.158)			0.811	(0.322, 1.300)		
Time effect		1.000		0.978	(0.951, 1.005)			0.978	(0.947, 1.008)		
Quadratic link function											
Environmental factors											
a-Tocopherol	1	0.560	1	0.558	(0.500, 0.617)	31.2	1	0.526	(0.288, 0.764)	0.614	(0.565, 0.664)
g-tocopherol	2	0.490	2	0.499	(0.453, 0.544)	24.9	3	0.333	(0.176, 0.489)	0.389	(0.324, 0.454)
Retinyl-palmitate	3	0.420	3	0.422	(0.363, 0.482)	17.8	4	0.279	(0.090, 0.467)	0.325	(0.242, 0.409)
Retinol	7	0.140	7	0.167	(0.108, 0.227)	2.8	8	−0.006	(−0.181, 0.169)	−0.007	(−0.078, 0.064)
3,3,4,4,5-pncb	8	0.070	8	0.073	(0.024, 0.122)	0.5	6	0.216	(0.027, 0.405)	0.252	(0.164, 0.341)
PCB194	6	−0.210	6	−0.209	(−0.269, − 0.149)	4.4	2	0.378	(0.137, 0.619)	0.441	(0.352, 0.531)
2.3.4.6.7.8.hxcdf	5	−0.280	5	−0.268	(−0.327, − 0.209)	7.2	7	− 0.061	(− 0.221, 0.100)	−0.071	(− 0.141, − 0.001)
trans.b.carotene	4	− 0.350	4	− 0.335	(− 0.388, − 0.283)	11.3	5	−0.273	(− 0.44, − 0.106)	−0.319	(− 0.381, − 0.257)
Covariates											
Intercept		0.000		0.877	(0.653, 1.100)			2.202	(1.478, 2.925)		
Age		0.005		0.007	(0.004, 0.009)			−0.023	(−0.035, −0.011)		
Sex (female)		−0.076		−0.061	(−0.158, 0.036)			−0.150	(−0.465, 0.165)		
Race/Ethnicity											
Non-Hispanic white		Ref		Ref				Ref			
Non-Hispanic black		−0.138		−0.078	(−0.206, 0.050)			−0.004	(−0.395, 0.387)		
Mexican American		0.175		0.219	(0.081, 0.358)			0.323	(−0.070, 0.717)		
Other race		0.409		0.642	(0.372, 0.911)			0.095	(−0.763, 0.953)		
Other Hispanic		0.355		0.152	(−0.093, 0.397)			−0.125	(−0.976, 0.726)		
Time effect		1.000		1.014	(0.987, 1.041)			1.013	(0.983, 1.044)		

### Performance evaluation

In all analyses, the estimated coefficients for the 8 environmental factors and confounders were reported. Ranks based on the absolute values of estimated coefficients were presented to evaluate the relative importance of each environmental factor, and squares of estimated coefficients were shown to represent the respective proportion of contribution to the single index. For all models, the standard errors of coefficient estimates and of the estimated link function were estimated using 500 runs of bootstrapping samples and used to construct the 95% confidence intervals (CIs). We compared the performance of each PLSI model with its counterpart parametric model. The estimated coefficients of 8 environmental factors from the parametric counterpart models were reported in both original values and scaled values to have *l*_2_ norm of 1 for comparison.

### Statistical software

All statistical analyses were performed using statistical software R 3.5.0. R codes for the PLSI models for different types of outcomes were developed using ‘gam’, ‘qgam’ or ‘gamm’ function call from ‘mgcv’ or ‘qgam’ package. Linear regression and logistic regression were fit using ‘glm’ function, and quantile regressions using ‘rq’ function in the ‘quantreg’ package. Cox PH model was fitted using ‘coxph’ function from ‘survival’ package, and linear mixed-effects model using ‘lme’ function from ‘nlme’ package. All descriptive and analytical codes were provided as an R Markdown document in Additional file 3.

## Results

### Continuous triglycerides: PLSI mean regression

**Fig. 2:**
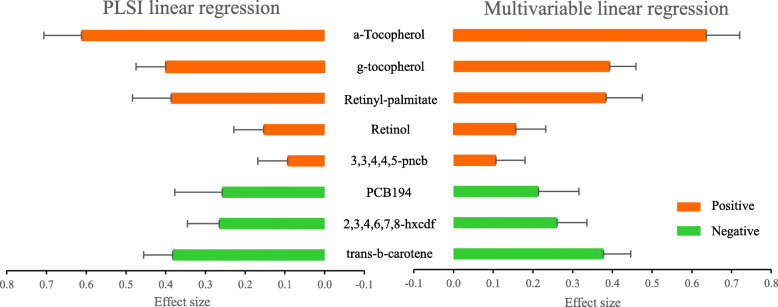
Results from PLSI linear regression and multivariable linear regression in NHANES 2002–2003 (d = 8, N = 800). Bars show the estimated relative importance (absolute value of estimated coefficient) of 8 environmental factors on continuous triglycerides. Red/green color represents positive/negative effect. Error bars indicate 95% CIs

We applied the PLSI linear regression and multivariable linear regression to study the associations of the 8 environmental factors with continuous triglycerides, and summarized the estimates in Fig. 2 (numerical results in Additional file 1: Table S2). The ranks, estimated coefficients, and directions were similar between these two models, and the estimated link function was close to be linear (Additional file 1: Figure S3). As the estimated link function was monotone and increasing, the positive estimates indicated a positive association with triglycerides. Specifically, a-Tocopherol had a ${\hat{\beta}}_1=0.612$ and 95% CI of (0.517, 0.707), indicating that a-Tocopherol had the strongest positive association with triglycerides among the 8 factors, and made about 37.4% contribution to the single index; trans-b-carotene had the most negative association of ${\hat{\beta}}_8=-0.383$. These results were consistent with original results from Patel’s study, which also observed a-Tocopherol with the strongest positive and trans-b-carotene with the strongest negative association with triglycerides [48]. As the 8 environmental factors showed both positive and negative associations with triglycerides, this application highlighted the need of statistical methods to accommodate both directional effects for studying multiple environmental exposures. Sensitivity analysis including all 22 environmental factors (Additional file 1: Table S3) showed that the conclusions on the important environmental factors were consistent. The 8 selected environmental factors consistently showed top ranks among the 22 factors, except for PCB194 which was highly correlated with other PCBs. When there are many highly correlated exposures (*r* > 0.9), we also recommend using *p*-values to rank the importance of variables in addition to the absolute coefficient values, which can be inflated by multicollinearity [65].

### Continuous triglycerides: PLSI quantile regression

**Fig. 3:**
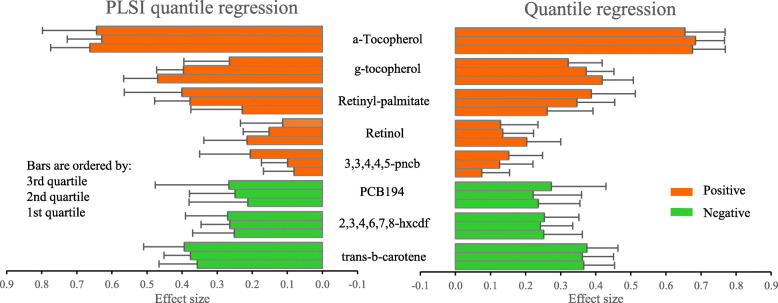
Results from PLSI quantile regression and multivariable quantile regression in NHANES 2002–2003 (d = 8, *N* = 800). Bars show the estimated relative importance (absolute value of estimated coefficient) of 8 environmental factors on three quartiles (25th, 50th, and 75th percentiles) of triglycerides. Red/green color represents positive/negative effect. Error bars indicate 95% CIs

We applied the PLSI quantile regression to study the associations between 8 exposures and three quartiles (25th, 50th, and 75th percentiles) of triglycerides and summarized the main results in Fig. 3 (numerical results in Additional file 1: Table S4). We observed that the estimated link functions for all three quartiles were increasing and close to be linear (Additional file 1: Figure S4), which explained the similarities between the results of the PLSI quantile regressions and regular quantile regressions. In addition, the 8 environmental factors showed fairly consistent associations across the three quartiles of triglycerides. For example, a-Tocopherol was the factor having the strongest positive association with triglycerides and trans-b-carotene was the factor having the strongest negative association with triglycerides at all three quartiles.

### Binary triglycerides: PLSI logistic regression

**Fig. 4:**
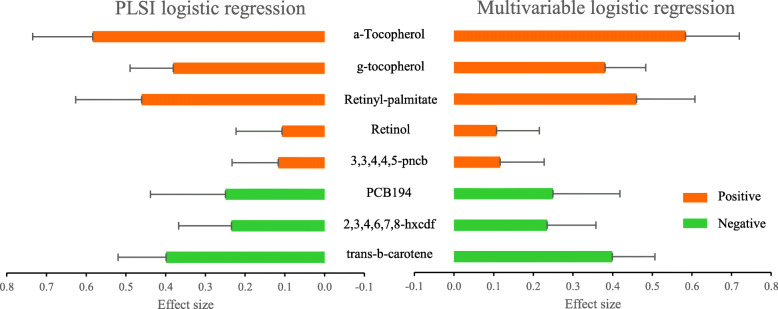
Results from PLSI logistic regression and multivariable logistic regression in NHANES 2002–2003 (N = 800). Bars show the estimated relative importance (absolute value of estimated coefficient) of 8 environmental factors on dichotomized triglycerides. Red/green color represents positive/negative effect. Error bars indicate 95% CIs

For dichotomized triglycerides, the ranks and estimates from PLSI logistic regression and multivariable logistic regression are shown in Fig. 4 (numerical results in Additional file 1: Table S5), which demonstrated similar results from these two models. The estimated link function by PLSI logistic regression was monotone increasing and close to be linear (Additional file 1: Figure S5). Thus, the estimated directions can be interpreted qualitatively and the estimated coefficients represented the relative importance of each exposure on the log odds of high triglycerides. For example, the estimated coefficient of a-Tocopherol was ${\hat{\beta}}_1=0.584$ (95% CI: 0.433–0.735), which represented that a-Tocopherol had the strongest positive association with the odds of high triglycerides among the 8 factors.

### Simulated time-to-event outcome: PLSI PH model

We summarize the simulation results from both PLSI PH model and Cox PH model in Table 3. Under the identity link function setting, results from the PLSI PH model and the conventional Cox PH model were very similar as expected, and both close to the true values. The PLSI PH model estimated the link function to be close to the true linear function (Additional file 1: Figure S6 (a)). Under the quadratic link function setting, results from the PLSI PH model were still consistent to true coefficients, but the conventional Cox PH model failed for most of the environmental factors because the linear model assumption was insufficient. The PLSI PH model also captured the U-shape and estimated the link function close to the true quadratic function (Additional file 1: Figure S6 (b)). Stratified plots (Additional file 1: Figure S7) showed that a-Tocopherol had different effects on the outcome when trans-b-carotene was set at its 10th, 50th, and 90th percentiles, indicating the existence of an interaction between a-Tocopherol and trans-b-carotene in this scenario. Results for complex polynomial link function (Additional file 1: Table S6 and Figure S8) presented good performance in coefficient and link function estimations, suggesting that PLSI models are able to handle complex higher-order interactions among environmental factors.

### Simulated longitudinal outcome: PLSI mixed-effects model

The results from PLSI mixed-effects model and linear mixed-effects model under identify or quadratic link function are presented in Table 4. Under the identity link function setting, the PLSI mixed-effects model estimated all coefficients close to the true coefficients with correct directions, and conventional linear mixed-effects model also had similar estimations. The estimated link function by PLSI mixed-effects model was close to the true linear function (Additional file 1: Figure S9 (a)). Under the quadratic link function setting, the results from PLSI mixed-effects model were still consistent; however, the conventional linear mixed-effects model clearly showed biased results for some factors like PCB194. The estimated link function by PLSI mixed-effects model had a U-shape and was close to the true quadratic function (Additional file 1: Figure S9 (b)).

## Discussion

We presented five PLSI models aiming to provide a unified family of statistical models to assess the joint effects of environmental exposures on four types of health outcomes: continuous, categorical, time-to-event, and longitudinal outcomes. We demonstrated the flexibility and effectiveness of this PLSI family for modeling various types of outcomes using NHANES data supplemented with simulations. One contribution of this work is that the novel modeling options under the PLSI framework complement existing methods and address some common statistical challenges in the analysis of multiple environmental exposures, such as mixed directions, interactions, and non-linear effects. Another contribution is that coherent computation algorithms are developed for all the PLSI models and implemented using the existing R packages, which can facilitate direct applications in practice and reproducible research.

In our analyses of the cross-sectional NHANES studies for continuous and binary triglycerides by PLSI models, we found that the 8 environmental factors exhibited mixed directional associations with the outcome, with a-Tocopherol having the strongest positive association and trans-b-carotene having the strongest negative association with triglycerides. A-Tocopherol and carotenes are transported in serum with HDL and LDL, and the level of serum a-Tocopherol depends on serum lipids [66, 67]. The strong positive association between a-Tocopherol and triglycerides is expected [48], and the negative association between b-carotene and triglycerides is supported by previous studies [68, 69]. Our results were consistent with the results of previously known and validated environmental chemical factors correlated with triglycerides [48], clearly demonstrating the value of PLSI models as a flexible and useful tool for analyzing complex exposures. Using additional simulations for time-to-event and longitudinal outcomes, we showed that the PLSI models could correctly identify the directions and magnitudes of associations for these environmental factors in scenarios with different types of outcomes.

In our NHANES applications of studying triglycerides continuously and categorically, we estimated that the link functions of PLSI models were very close to be linear, which were also reflected by the similar results with their counterpart parametric models. In general, standard errors from the PLSI models were larger than those from their counterpart parametric models, which was expected as the former are semiparametric models.

We also conducted another sensitivity weighted analysis incorporating the laboratory subsample C weights from NHANES 2003–2004 cycle (following general guideline to use the weights from “least common denominator”) [70], and the weighted results (Additional file 1: Table S7) were similar with the results from unweighted models. Note that most of the PLSI models are readily incorporate weights in R function codes (Additional file 3).

Interaction among multiple correlated environmental factors is very common, and it has been long appreciated that the co-exposures may have synergistic (additive or multiplicative) or antagonistic effects on health outcomes [71]. For parametric models, it’s difficult to directly model the interaction effects among co-exposures if we don’t know the ‘degree of interaction’. However, PLSI models can handle the interaction easily through the unknown link function as we evaluated using the simulations. Specifically, in our simulated time-to-event and longitudinal analyses with quadratic link function , which reflected both the pairwise interactions and non-linear quadratic effects, both PLSI PH model and PLSI mixed-effects were able to capture the U-shape link function and correct direction and importance of the environmental factors, while parametric models failed in most factors because the parametric assumptions were no longer satisfied. For more complex (higher-order) interactions, the flexibility of the nonparametric link function can incorporate the effects of these interactions [72]. Therefore, PLSI models readily accommodate the factors showing non-linear or interactive effect on the health outcome.

There are other ways and models using various definitions of weighted sums to model the joint effect for multiple environmental components. For example, molar sums were used to show relationships between prenatal phenol and phthalate exposures and birth outcome [73], and a potency-weighted sum was used to calculate phthalates exposures among reproductive-aged women [74]. The weights for environmental factors can be calculated from their expected potency relative to a reference factor, like the common cases in toxicology [75], or based on their percent contribution to the total mixture effect, like WQS [9]. PLSI models can be considered as one of these weighting approaches, and their advantages from the semiparametric structure are evident compared with existing methods, especially for the scenarios when the environmental exposures have mixed-directional associations and/or a potential high-degree interaction. Meanwhile, due to the flexibility of the nonparametric link function, PLSI models can represent complex joint effects more than additive structures [76], which is commonly encountered since environmental exposures may act together in a biological sense via a shared mechanistic pathway [4]. The ability of handling various types of outcomes is another important advantage of the proposed PLSI framework. This is important because, with the accumulation of environmental exposure measurements and development of data collection methods, time-to-event or longitudinal studies are desired to explore the associations over time.

In this study, the coherent algorithms for PLSI models are based on the ‘gam’ and ‘gamm’ functions from ‘mgcv’ package and ‘qgam’ function from ‘qgam’ package in R, which includes many of the generalized additive model (GAM) fitting techniques developed by Simon Wood et al. [77]. The rationale behind the algorithms is to use ‘gam’, ‘qgam’ or ‘gamm’ call (usually using penalized regression splines or similar smoothers) to profile out the smooth model coefficients and smoothing parameters for estimation of the link function contained in PLSI model, leaving only a finite parameter vector to be estimated by a general purpose optimizer. Based on this algorithm, it is easy to adapt the models to include multiple single index terms, parametric terms, and further smoothing. We have compared the estimates for single index models among different iterative procedures using existing packages (e.g., projection pursuit regression with one term using ‘ppr’ function; ‘sim.est’ function from ‘simest’ package) in various simulations, and they have similar estimation performance. We finally chose ‘gam’ call series because of its flexibility for covariate adjustment and ability of modeling various types of outcomes. This ‘gam’, ‘qgam’, ‘gamm’ call approach has demonstrated efficient and robust performance in our numerical studies, and we believe this coherent algorithm strategy wrapped as a toolbox is beneficial for practical application.

The PLSI models considered here may not be directly applicable to extreme high-dimensional settings, for which we could consider using extensions with adaptive LASSO [78], smoothly clipped absolute deviation penalty [79], and smooth-threshold estimating equations [80]. Another future research direction is to extend from the single index to multiple-index models, such as the projection pursuit regression [81], so that more complex data structures and exposure effect patterns can be captured and modeled.

## Conclusions

A family of PLSI models exemplified great value of identifying important components among environmental exposures when they demonstrate associations in various directions and complex non-linear relationships between the exposures and outcome.

## Supplementary information

**Additional file 1: Figure S1.** Data flow diagram for deriving 800 subjects and 8 environmental factors. **Figure S2.** Correlation matrix of Pearson correlation coefficient of 22 factors and triglycerides in NHANES 2002–2003 (*N* = 800). **Table S1.** PLSI generalized linear regression for ordinal, multinomial, and count outcomes and PLSI mixed-effects model with random slope for longitudinal outcome. **Table S2.** Results from PLSI linear regression and multivariable linear regression in NHANES 2002–2003. **Figure S3.** Estimated link function by PLSI linear regression in NHANES 2002–2003. **Table S3.** Sensitivity analysis results from PLSI linear regression and multivariable linear regression in NHANES 2002–2003 with 22 environmental factors. **Tables S4.1-S4.3.** Results from PLSI quantile regressions and multivariable quantile regression at three quantiles (25th, 50th, and 75th percentiles) of triglycerides in NHANES 2002–2003. **Figure S4.** Estimated link functions by PLSI quantile regressions at three quartiles in NHANES 2002–2003. (a) 25th percentile; (b) 50th percentile; (c) 75th percentile. **Table S5.** Results from PLSI logistic regression and multivariable logistic regression in NHANES 2002. **Figure S5.** Estimated link function by PLSI logistic regression in NHANES 2002–2003. **Figure S6.** Estimated link functions by PLSI PH model in simulated time-to-event study. (a) identity link function; (b) quadratic link function. **Figure S7.** Stratified effect of a-Tocopherol with 95% confidence intervals when the variable of trans-b-carotene fixed at 10, 50, and 90 percentile and other factors fixed as median values. **Table S6.** Simulation results from PLSI PH model and Cox PH model for link function *g*(*x*) = 0.2*x*^3^ − *x*^2^ + 3*x*. **Figure S8.** Estimated link functions by PLSI PH model in simulated time-to-event study with link function *g*(*x*) = 0.2*x*^3^ − *x*^2^ + 3*x*. **Figure S9.** Estimated link functions by PLSI mixed-effects model in simulated longitudinal study. (a) identity link function; (b) quadratic link function. **Table S7.** Sensitivity analysis results from weighted PLSI linear regression and weighted linear regression in NHANES 2002–2003 using NHANES laboratory subsample C weights.

**Additional file 2.** Cleaning dataset of 800 subjects from NHANES 2003–2004 cycle. Variables include respondent sequence number of subject, outcome triglyceride, 22 environmental factors, 3 demographic confounding variables, and laboratory subsample C weight.

**Additional file 3.** R markdown document demonstrating all descriptive and analytical process of this article.

## Data Availability

The dataset used and/or analyzed during the current study supporting the conclusions of this article is included within the additional file.

## Abbreviations

AR: Autoregressive process
BKMR: Bayesian kernel machine regression
NHANES: National Health and Nutrition Examination Survey
NIEHS: National Institute of Environmental Health Sciences
PH: Proportional hazards
PLSI: Partial-linear single-index
PIP: Posterior inclusion probability
QR: Quantile regression
WQS: Weighted quantile sum regression
