# Supplementary material for: A family of partial-linear single-index models for analyzing complex environmental exposures with continuous, categorical, time-to-event, and longitudinal health outcomes
Source: Environ Health. 2020 Sep 11;19:96. doi: 10.1186/s12940-020-00644-4 (PMC7488560; doi:10.1186/s12940-020-00644-4)
Supplement: Supplementary file 1 — Additional file 1: Figure S1. Data flow diagram for deriving 800 subjects and 8 environmental factors. Figure S2. Correlation matrix of Pearson correlation coefficient of 22 factors and triglycerides in NHANES 2002–2003 (N = 800). Table S1. PLSI generalized linear regression for ordinal, multinomial, and count outcomes and PLSI mixed-effects model with random slope for longitudinal outcome. Table S2. Results from PLSI linear regression and multivariable linear regression in NHANES 2002–2003. Figure S3. Estimated link function by PLSI linear regression in NHANES 2002–2003. Table S3. Sensitivity analysis results from PLSI linear regression and multivariable linear regression in NHANES 2002–2003 with 22 environmental factors. Tables S4.1-S4.3. Results from PLSI quantile regressions and multivariable quantile regression at three quantiles (25th, 50th, and 75th percentiles) of triglycerides in NHANES 2002–2003. Figure S4. Estimated link functions by PLSI quantile regressions at three quartiles in NHANES 2002–2003. (a) 25th percentile; (b) 50th percentile; (c) 75th percentile. Table S5. Results from PLSI logistic regression and multivariable logistic regression in NHANES 2002. Figure S5. Estimated link function by PLSI logistic regression in NHANES 2002–2003. Figure S6. Estimated link functions by PLSI PH model in simulated time-to-event study. (a) identity link function; (b) quadratic link function. Figure S7. Stratified effect of a-Tocopherol with 95% confidence intervals when the variable of trans-b-carotene fixed at 10, 50, and 90 percentile and other factors fixed as median values. Table S6. Simulation results from PLSI PH model and Cox PH model for link function g(x) = 0.2x3 − x2 + 3x. Figure S8. Estimated link functions by PLSI PH model in simulated time-to-event study with link function g(x) = 0.2x3 − x2 + 3x. Figure S9. Estimated link functions by PLSI mixed-effects model in simulated longitudinal study. (a) identity link function; (b) quadratic link function. Ta [file 12940_2020_644_MOESM1_ESM.docx]

**Additional files**

**A family of partial-linear single-index models for analyzing complex environmental exposures with continuous, categorical, time-to-event, and longitudinal health outcomes**

Yuyan Wang, Yinxiang Wu, Melanie Jacobson, Myeonggyun Lee, Peng Jin, Leonardo Trasande, Mengling Liu

**Table of Contents**

**Additional file 1: Additional Tables and Figures**

Figure S1. Data flow diagram for deriving 800 subjects and 8 environmental factors.

Figure S2. Correlation matrix of Pearson correlation coefficient of 22 factors and triglycerides in NHANES 2002-2003 (N=800).

Table S1. PLSI generalized linear regression for ordinal, multinomial, and count outcomes and PLSI mixed-effects model with random slope for longitudinal outcome.

Table S2. Results from PLSI linear regression and multivariable linear regression in NHANES 2002-2003.

Figure S3. Estimated link function by PLSI linear regression in NHANES 2002-2003.

Table S3. Sensitivity analysis results from PLSI linear regression and multivariable linear regression in NHANES 2002-2003 with 22 environmental factors.

Tables S4.1-S4.3. Results from PLSI quantile regressions and multivariable quantile regression at three quantiles (25th, 50th, and 75th percentiles) of triglycerides in NHANES 2002-2003.

Figure S4. Estimated link functions by PLSI quantile regressions at three quartiles in NHANES 2002-2003. (a) 25th percentile; (b) 50th percentile; (c) 75th percentile.

Table S5. Results from PLSI logistic regression and multivariable logistic regression in NHANES 2002.

Figure S5. Estimated link function by PLSI logistic regression in NHANES 2002-2003.

Figure S6. Estimated link functions by PLSI PH model in simulated time-to-event study. (a) identity link function; (b) quadratic link function.

Figure S7. Stratified effect of a-Tocopherol with 95% confidence intervals when the variable of trans-b-carotene fixed at 10%, 50%, and 90% percentile and other factors fixed as median values.

Table S6. Simulation results from PLSI PH model and Cox PH model for link function $g\left( x \right)=0.2x^{3}-x^{2}+3x$.

Figure S8. Estimated link functions by PLSI PH model in simulated time-to-event study with link function $g\left( x \right)=0.2x^{3}-x^{2}+3x$.

Figure S9. Estimated link functions by PLSI mixed-effects model in simulated longitudinal study. (a) identity link function; (b) quadratic link function.

Table S7. Sensitivity analysis results from weighted PLSI linear regression and weighted linear regression in NHANES 2002-2003 using NHANES laboratory subsample C weights.

**Additional file 2: cleaning dataset of 800 subjects from NHANES 2003-2004 cycle.**

Variables include respondent sequence number of subject, outcome triglyceride, 22 environmental factors, 3 demographic confounding variables, and laboratory subsample C weight.

**Additional file 3: R markdown document demonstrating all descriptive and analytical process of this article.**

**Additional file 1: Additional Tables and Figures**

10,122 people interviewed in NHANES 2003-2004 cycle

3,680 participants had serum triglyceride values

800 subjects were included in the final study

*Check outcome variable*

*Check other variables and delete subjects with missing values*

22 of 29 environmental factors in Patel’s paper were measured in the same overlapping individuals in NHANES 2003-2004 cycle

8 environmental factors were included for analyses

1. *Pearson correlation matrix found many correlation coefficients >0.95 (Additional file 1: Figure S2)*

2. *Multivariable linear regression using stepwise variables selection*

Figure S1. Data flow diagram for deriving 800 subjects and 8 environmental factors.


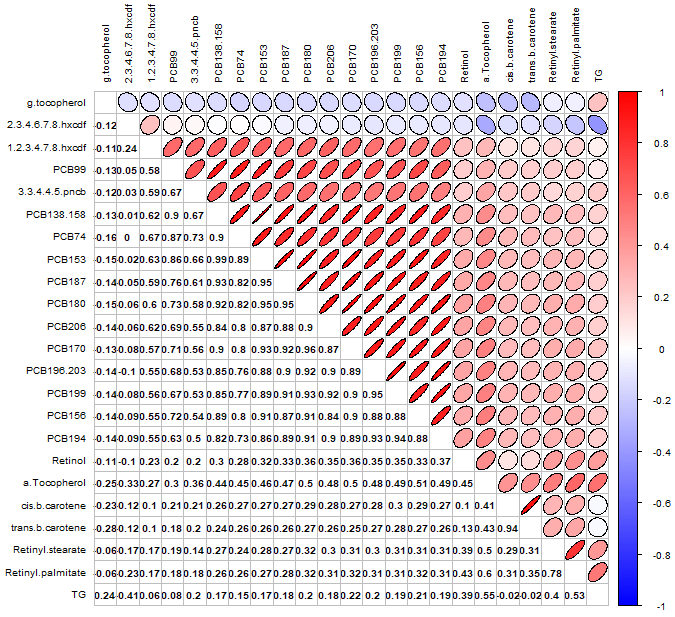


Figure S2. Correlation matrix of Pearson correlation coefficient of 22 factors and triglycerides in NHANES 2002-2003 (N=800).

Table S1. PLSI generalized linear regression for ordinal, multinomial, and count outcomes and PLSI mixed-effects model with random slope for longitudinal outcome.

| Outcome | Distribution | PLSI model specification |
| --- | --- | --- |
| Ordinal | $Y=\left( Y_{1},\ldots,Y_{C} \right)\sim Multinomial(n,p_{1},\ldots,p_{c-1})$ | $logit\left( P\left( Y\leq Y_{l}\left\vert X,Z \right. \right) \right)=\alpha_{l}+g\left( \sum_{j=1}^{d} \beta_{j}X_{j} \right)+\gamma^{'}Z$  $l\in\{1,2,\ldots,C-1\}$ |
| Multinomial | $Y=\left( Y_{1},\ldots,Y_{C} \right)\sim Multinomial(n,p_{1},\ldots,p_{c-1})$ | $log\left( \frac{P\left( Y=Y_{l}\left\vert X,Z \right. \right)}{P\left( Y=Y_{1}\left\vert X,Z \right. \right)} \right)=\alpha_{l}+g_{l}\left( \sum_{j=1}^{d} \beta_{jl}X_{j} \right)+\gamma_{l}^{'}Z$  $l\in\{2,\ldots,C\}$ |
| Count | $Y\sim Poisson(\lambda)$ | $log(E\left( Y\left\vert X,Z \right. \right))=g\left( \sum_{j=1}^{d} \beta_{j}X_{j} \right)+\gamma^{'}Z$ |
| Longitudinal | $Y\sim Normal;$ $b_{i}:$ random intercept; $d_{i}$: random slope | $Y_{ij}= g\left( \sum_{j=1}^{d} \beta_{j}X_{j} \right)+Z_{ij}^{'}\gamma+b_{i}+{(\omega+d_{i})T}_{ij}+\varepsilon_{ij}$ |

Table S2. Results from PLSI linear regression and multivariable linear regression in NHANES 2002-2003.

| Variable | PLSI LR rank | PLSI LR estimate | PLSI  LR 95% CI | PLSI LR Proportion of contribution (%) |  | LR rank | LR original estimate | LR original 95% CI | LR normed estimate | LR normed 95% CI |
| --- | --- | --- | --- | --- | --- | --- | --- | --- | --- | --- |
| Environmental factors |  |  |  |  |  |  |  |  |  |  |
| a-Tocopherol | 1 | 0.612 | (0.517, 0.707) | 37.4 |  | 1 | 0.459 | (0.388, 0.530) | 0.636 | (0.552, 0.721) |
| g-tocopherol | 2 | 0.400 | (0.326, 0.475) | 16.0 |  | 2 | 0.284 | (0.237, 0.330) | 0.393 | (0.327, 0.459) |
| Retinyl-palmitate | 3 | 0.386 | (0.289, 0.484) | 14.9 |  | 3 | 0.277 | (0.220, 0.334) | 0.384 | (0.293, 0.475) |
| Retinol | 7 | 0.154 | (0.080, 0.228) | 2.4 |  | 7 | 0.113 | (0.061, 0.166) | 0.157 | (0.082, 0.232) |
| 3,3,4,4,5-pncb | 8 | 0.093 | (0.018, 0.168) | 0.9 |  | 8 | 0.077 | (0.020, 0.133) | 0.107 | (0.033, 0.180) |
| PCB194 | 6 | -0.258 | (-0.377, -0.138) | 6.6 |  | 6 | -0.155 | (-0.227, -0.082) | -0.215 | (-0.316, -0.113) |
| 2.3.4.6.7.8.hxcdf | 5 | -0.266 | (-0.345, -0.186) | 7.1 |  | 5 | -0.188 | (-0.236, -0.140) | -0.261 | (-0.336, -0.186) |
| trans.b.carotene | 4 | -0.383 | (-0.456, -0.310) | 14.7 |  | 4 | -0.273 | (-0.323, -0.222) | -0.378 | (-0.447, -0.309) |
| Covariates |  |  |  |  |  |  |  |  |  |  |
| Intercept |  | -0.128 | (-0.375, 0.119) |  |  |  | -0.051 | (-0.266, 0.165) |  |  |
| Age |  | 0.005 | (0.001, 0.010) |  |  |  | 0.003 | (0.000, 0.007) |  |  |
| Sex (female) |  | -0.076 | (-0.167, 0.016) |  |  |  | -0.077 | (-0.172, 0.018) |  |  |
| Ethnicity |  |  |  |  |  |  |  |  |  |  |
| Non-Hispanic white |  | Ref |  |  |  |  | Ref |  |  |  |
| Non-Hispanic black |  | -0.138 | (-0.264, -0.011) |  |  |  | -0.126 | (-0.243, -0.008) |  |  |
| Mexican American |  | 0.175 | (0.054, 0.297) |  |  |  | 0.188 | (0.069, 0.307) |  |  |
| Other race |  | 0.409 | (0.142, 0.676) |  |  |  | 0.383 | (0.128, 0.638) |  |  |
| Other Hispanic |  | 0.355 | (0.083, 0.627) |  |  |  | 0.355 | (0.098, 0.612) |  |  |


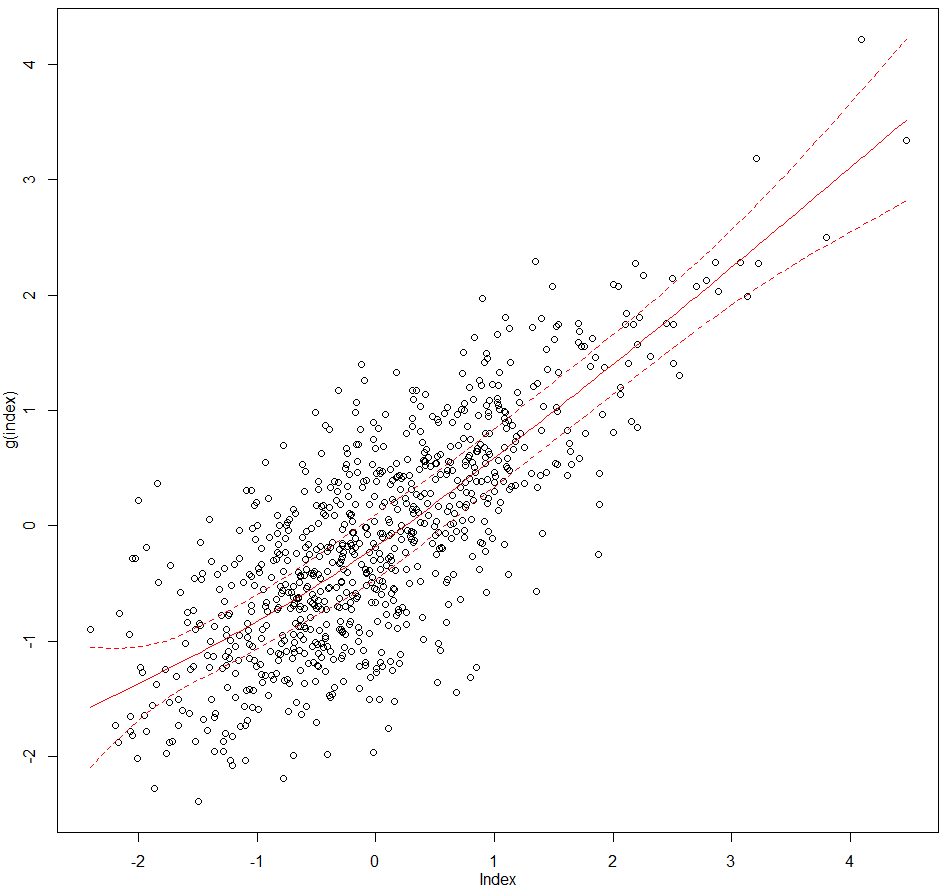


Figure S3. Estimated link function by PLSI linear regression in NHANES 2002-2003.

Table S3. Sensitivity analysis results from PLSI linear regression and multivariable linear regression in NHANES 2002-2003 with 22 environmental factors.

| Variable | PLSI LR absolute estimate rank | PLSI LR estimate | PLSI LR SE | PLSI LR p value | PLSI LR p value rank |  | LR absolute estimate rank | LR original estimate | LR original SE | LR normed estimate | LR normed SE | LR p value | LR p value rank |
| --- | --- | --- | --- | --- | --- | --- | --- | --- | --- | --- | --- | --- | --- |
| Environmental factors |  |  |  |  |  |  |  |  |  |  |  |  |  |
| a-Tocopherol | 1 | 0.475 | 0.0888 | 1.16E-07 | 2 |  | 1 | 0.441 | 0.0370 | 0.509 | 0.0680 | 3.34E-30 | 1 |
| PCB138.158 | 3 | 0.375 | 0.2087 | 7.31E-02 | 10 |  | 3 | 0.329 | 0.1868 | 0.380 | 0.1954 | 7.86E-02 | 9 |
| Retinyl-palmitate | 4 | 0.337 | 0.0650 | 2.73E-07 | 3 |  | 4 | 0.281 | 0.0391 | 0.325 | 0.0494 | 1.50E-12 | 3 |
| g-tocopherol | 5 | 0.314 | 0.0583 | 9.78E-08 | 1 |  | 5 | 0.274 | 0.0241 | 0.317 | 0.0427 | 7.88E-28 | 2 |
| 3.3.4.4.5.pncb | 9 | 0.147 | 0.0453 | 1.18E-03 | 7 |  | 8 | 0.133 | 0.0364 | 0.154 | 0.0412 | 2.70E-04 | 7 |
| PCB170 | 10 | 0.139 | 0.1209 | 2.51E-01 | 13 |  | 9 | 0.129 | 0.0858 | 0.149 | 0.0936 | 1.34E-01 | 12 |
| Retinol | 11 | 0.125 | 0.0334 | 1.92E-04 | 5 |  | 11 | 0.109 | 0.0269 | 0.126 | 0.0310 | 5.25E-05 | 5 |
| PCB196.203 | 14 | 0.088 | 0.0855 | 3.04E-01 | 14 |  | 16 | 0.049 | 0.0804 | 0.056 | 0.0753 | 5.44E-01 | 15 |
| PCB187 | 18 | 0.052 | 0.1080 | 6.28E-01 | 17 |  | 20 | 0.022 | 0.0904 | 0.025 | 0.0921 | 8.10E-01 | 21 |
| cis.b.carotene | 22 | -0.007 | 0.0856 | 9.36E-01 | 22 |  | 19 | -0.029 | 0.0627 | -0.034 | 0.0842 | 6.42E-01 | 18 |
| Retinyl-stearate | 21 | -0.011 | 0.0410 | 7.90E-01 | 21 |  | 21 | 0.009 | 0.0365 | 0.011 | 0.0400 | 7.99E-01 | 20 |
| PCB199 | 20 | -0.036 | 0.0975 | 7.13E-01 | 19 |  | 22 | 0.004 | 0.0881 | 0.004 | 0.0921 | 9.66E-01 | 22 |
| PCB206 | 19 | -0.039 | 0.0857 | 6.51E-01 | 18 |  | 17 | -0.037 | 0.0666 | -0.043 | 0.0790 | 5.79E-01 | 16 |
| 1.2.3.4.7.8.hxcdf | 17 | -0.064 | 0.0368 | 8.25E-02 | 11 |  | 15 | -0.050 | 0.0332 | -0.058 | 0.0363 | 1.34E-01 | 11 |
| PCB156 | 16 | -0.078 | 0.0824 | 3.43E-01 | 16 |  | 18 | -0.033 | 0.0655 | -0.038 | 0.0747 | 6.16E-01 | 17 |
| PCB153 | 15 | -0.083 | 0.2471 | 7.38E-01 | 20 |  | 14 | -0.077 | 0.2167 | -0.089 | 0.2386 | 7.22E-01 | 19 |
| PCB194 | 13 | -0.092 | 0.0931 | 3.24E-01 | 15 |  | 13 | -0.085 | 0.0742 | -0.098 | 0.0779 | 2.53E-01 | 14 |
| PCB99 | 12 | -0.094 | 0.0752 | 2.14E-01 | 12 |  | 12 | -0.093 | 0.0719 | -0.107 | 0.0699 | 1.96E-01 | 13 |
| PCB74 | 8 | -0.157 | 0.0736 | 3.30E-02 | 9 |  | 10 | -0.120 | 0.0691 | -0.139 | 0.0700 | 8.27E-02 | 10 |
| 2.3.4.6.7.8.hxcdf | 7 | -0.194 | 0.0420 | 4.53E-06 | 4 |  | 7 | -0.170 | 0.0260 | -0.197 | 0.0338 | 1.10E-10 | 4 |
| trans.b.carotene | 6 | -0.302 | 0.0815 | 2.20E-04 | 6 |  | 6 | -0.245 | 0.0638 | -0.283 | 0.0816 | 1.31E-04 | 6 |
| PCB180 | 2 | -0.405 | 0.1312 | 2.10E-03 | 8 |  | 2 | -0.335 | 0.1234 | -0.387 | 0.1255 | 6.73E-03 | 8 |
| Covariates |  |  |  |  |  |  |  |  |  |  |  |  |  |
| Intercept |  | -0.265 | 0.1334 | 4.70E-02 |  |  |  | -0.174 | 0.1239 |  |  | 1.61E-01 |  |
| Age |  | 0.009 | 0.0024 | 1.61E-04 |  |  |  | 0.007 | 0.0022 |  |  | 3.26E-03 |  |
| Sex (female) |  | -0.058 | 0.0501 | 2.44E-01 |  |  |  | -0.065 | 0.0505 |  |  | 1.99E-01 |  |
| Ethnicity |  |  |  |  |  |  |  |  |  |  |  |  |  |
| Non-Hispanic white |  | Ref |  |  |  |  |  | Ref |  |  |  |  |  |
| Non-Hispanic black |  | -0.157 | 0.0688 | 2.24E-02 |  |  |  | -0.137 | 0.0635 |  |  | 3.09E-02 |  |
| Mexican American |  | 0.087 | 0.0673 | 1.98E-01 |  |  |  | 0.116 | 0.0650 |  |  | 7.54E-02 |  |
| Other race |  | 0.316 | 0.1485 | 3.36E-02 |  |  |  | 0.311 | 0.1354 |  |  | 2.20E-02 |  |
| Other Hispanic |  | 0.316 | 0.1541 | 4.09E-02 |  |  |  | 0.322 | 0.1341 |  |  | 1.67E-02 |  |

Table S4-1. Results from PLSI quantile regressions and multivariable quantile regression at the 25th quantile of triglycerides in NHANES 2002-2003.

| Percentile | Variable | PLSI QR rank | PLSI QR estimate | PLSI  QR 95% CI | PLSI QR Proportion of contribution (%) |  | QR rank | QR original estimate | QR original 95% CI | QR normed estimate | QR normed 95% CI |
| --- | --- | --- | --- | --- | --- | --- | --- | --- | --- | --- | --- |
| 25 | Environmental factors |  |  |  |  |  |  |  |  |  |  |
|  | a-Tocopherol | 1 | 0.663 | (0.552, 0.774) | 44.0 |  | 1 | 0.527 | (0.415, 0.638) | 0.676 | (0.582, 0.769) |
|  | g-tocopherol | 2 | 0.470 | (0.373, 0.566) | 22.0 |  | 2 | 0.326 | (0.249, 0.403) | 0.418 | (0.329, 0.507) |
|  | Retinyl-palmitate | 5 | 0.228 | (0.082, 0.375) | 5.2 |  | 4 | 0.203 | (0.111, 0.296) | 0.261 | (0.130, 0.392) |
|  | Retinol | 6 | 0.215 | (0.091, 0.338) | 4.6 |  | 7 | 0.158 | (0.084, 0.233) | 0.203 | (0.107, 0.300) |
|  | 3,3,4,4,5-pncb | 8 | 0.081 | (-0.006, 0.168) | 0.7 |  | 8 | 0.058 | (-0.004, 0.121) | 0.075 | (-0.004, 0.155) |
|  | PCB194 | 7 | -0.212 | (-0.380, -0.044) | 4.5 |  | 6 | -0.184 | (-0.280, -0.088) | -0.236 | (-0.355, -0.118) |
|  | 2.3.4.6.7.8.hxcdf | 4 | -0.251 | (-0.370, -0.132) | 6.3 |  | 5 | -0.196 | (-0.272, -0.120) | -0.252 | (-0.362, -0.142) |
|  | trans.b.carotene | 3 | -0.356 | (-0.466, -0.247) | 12.7 |  | 3 | -0.285 | (-0.361, -0.209) | -0.366 | (-0.454, -0.278) |
|  | Covariates |  |  |  |  |  |  |  |  |  |  |
|  | Intercept |  | -0.589 | (-0.868, -0.309) |  |  |  | -0.521 | (-0.783, -0.259) |  |  |
|  | Age |  | 0.005 | (0.000, 0.010) |  |  |  | 0.004 | (0.000, 0.009) |  |  |
|  | Sex (female) |  | -0.051 | (-0.164, 0.063) |  |  |  | -0.040 | (-0.161, 0.081) |  |  |
|  | Ethnicity |  |  |  |  |  |  |  |  |  |  |
|  | Non-Hispanic white |  | Ref |  |  |  |  | Ref |  |  |  |
|  | Non-Hispanic black |  | -0.150 | (-0.292, -0.007) |  |  |  | -0.182 | (-0.340, -0.023) |  |  |
|  | Mexican American |  | 0.187 | (0.044, 0.329) |  |  |  | 0.131 | (-0.016, 0.279) |  |  |
|  | Other race |  | 0.442 | (0.022, 0.862) |  |  |  | 0.526 | (-0.010, 1.062) |  |  |
|  | Other Hispanic |  | 0.336 | (0.019, 0.652) |  |  |  | 0.238 | (-0.110, 0.586) |  |  |

Table S4-2. Results from PLSI quantile regressions and multivariable quantile regression at the 50th quantile of triglycerides in NHANES 2002-2003.

| Percentile | Variable | PLSI QR rank | PLSI QR estimate | PLSI  QR 95% CI | PLSI QR Proportion of contribution (%) |  | QR rank | QR original estimate | QR original 95% CI | QR normed estimate | QR normed 95% CI |
| --- | --- | --- | --- | --- | --- | --- | --- | --- | --- | --- | --- |
| 50 | Environmental factors |  |  |  |  |  |  |  |  |  |  |
|  | a-Tocopherol | 1 | 0.629 | (0.530, 0.727) | 39.5 |  | 1 | 0.523 | (0.434, 0.613) | 0.684 | (0.582, 0.769) |
|  | g-tocopherol | 2 | 0.396 | (0.319, 0.472) | 15.7 |  | 2 | 0.285 | (0.220, 0.350) | 0.373 | (0.329, 0.507) |
|  | Retinyl-palmitate | 3 | 0.377 | (0.276, 0.478) | 14.2 |  | 4 | 0.265 | (0.189, 0.341) | 0.346 | (0.130, 0.392) |
|  | Retinol | 7 | 0.152 | (0.079, 0.226) | 2.3 |  | 7 | 0.103 | (0.036, 0.171) | 0.135 | (0.107, 0.300) |
|  | 3,3,4,4,5-pncb | 8 | 0.099 | (0.024, 0.174) | 1.0 |  | 8 | 0.096 | (0.022, 0.171) | 0.126 | (-0.004, 0.155) |
|  | PCB194 | 6 | -0.249 | (-0.379, -0.120) | 6.2 |  | 6 | -0.170 | (-0.282, -0.058) | -0.222 | (-0.355, -0.118) |
|  | 2.3.4.6.7.8.hxcdf | 5 | -0.264 | (-0.346, -0.182) | 7.0 |  | 5 | -0.185 | (-0.250, -0.120) | -0.242 | (-0.362, -0.142) |
|  | trans.b.carotene | 4 | -0.376 | (-0.452, -0.300) | 14.1 |  | 3 | -0.277 | (-0.354, -0.200) | -0.362 | (-0.454, -0.278) |
|  | Covariates |  |  |  |  |  |  |  |  |  |  |
|  | Intercept |  | -0.119 | (-0.379, 0.141) |  |  |  | -0.039 | (-0.332, 0.253) |  |  |
|  | Age |  | 0.004 | (-0.001, 0.009) |  |  |  | 0.002 | (-0.003, 0.006) |  |  |
|  | Sex (female) |  | -0.064 | (-0.161, 0.033) |  |  |  | -0.034 | (-0.161, 0.092) |  |  |
|  | Ethnicity |  |  |  |  |  |  |  |  |  |  |
|  | Non-Hispanic white |  | Ref |  |  |  |  | Ref |  |  |  |
|  | Non-Hispanic black |  | -0.121 | (-0.255, 0.013) |  |  |  | -0.130 | (-0.291, 0.030) |  |  |
|  | Mexican American |  | 0.198 | (0.070, 0.326) |  |  |  | 0.199 | (0.047, 0.351) |  |  |
|  | Other race |  | 0.460 | (0.175, 0.745) |  |  |  | 0.514 | (0.193, 0.834) |  |  |
|  | Other Hispanic |  | 0.361 | (0.067, 0.656) |  |  |  | 0.367 | (-0.045, 0.779) |  |  |

Table S4-3. Results from PLSI quantile regressions and multivariable quantile regression at the 75th quantile of triglycerides in NHANES 2002-2003.

| Percentile | Variable | PLSI QR rank | PLSI QR estimate | PLSI  QR 95% CI | PLSI QR Proportion of contribution (%) |  | QR rank | QR original estimate | QR original 95% CI | QR normed estimate | QR normed 95% CI |
| --- | --- | --- | --- | --- | --- | --- | --- | --- | --- | --- | --- |
| 75 | Environmental factors |  |  |  |  |  |  |  |  |  |  |
|  | a-Tocopherol | 1 | 0.644 | (0.490, 0.798) | 41.5 |  | 1 | 0.474 | (0.359, 0.589) | 0.654 | (0.539, 0.769) |
|  | g-tocopherol | 6 | 0.265 | (0.135, 0.395) | 7.0 |  | 4 | 0.233 | (0.161, 0.305) | 0.321 | (0.224, 0.418) |
|  | Retinyl-palmitate | 2 | 0.400 | (0.236, 0.565) | 16.0 |  | 2 | 0.281 | (0.197, 0.365) | 0.388 | (0.263, 0.513) |
|  | Retinol | 8 | 0.113 | (-0.007, 0.234) | 1.3 |  | 8 | 0.093 | (0.016, 0.170) | 0.129 | (0.022, 0.235) |
|  | 3,3,4,4,5-pncb | 7 | 0.206 | (0.061, 0.350) | 4.2 |  | 7 | 0.110 | (0.037, 0.184) | 0.152 | (0.056, 0.248) |
|  | PCB194 | 5 | -0.267 | (-0.477, -0.057) | 7.1 |  | 5 | -0.198 | (-0.320, -0.076) | -0.274 | (-0.429, -0.118) |
|  | 2.3.4.6.7.8.hxcdf | 4 | -0.271 | (-0.391, -0.151) | 7.3 |  | 6 | -0.184 | (-0.255, -0.113) | -0.253 | (-0.352, -0.155) |
|  | trans.b.carotene | 3 | -0.394 | (-0.510, -0.278) | 15.5 |  | 3 | -0.272 | (-0.341, -0.203) | -0.375 | (-0.463, -0.286) |
|  | Covariates |  |  |  |  |  |  |  |  |  |  |
|  | Intercept |  | 0.469 | (0.077, 0.861) |  |  |  | 0.411 | (0.050, 0.772) |  |  |
|  | Age |  | 0.001 | (-0.006, 0.009) |  |  |  | 0.003 | (-0.002, 0.007) |  |  |
|  | Sex (female) |  | -0.097 | (-0.235, 0.040) |  |  |  | -0.087 | (-0.232, 0.058) |  |  |
|  | Ethnicity |  |  |  |  |  |  |  |  |  |  |
|  | Non-Hispanic white |  | Ref |  |  |  |  | Ref |  |  |  |
|  | Non-Hispanic black |  | -0.077 | (-0.25, 0.096) |  |  |  | -0.133 | (-0.308, 0.041) |  |  |
|  | Mexican American |  | 0.209 | (0.055, 0.363) |  |  |  | 0.202 | (0.047, 0.356) |  |  |
|  | Other race |  | 0.320 | (0.013, 0.627) |  |  |  | 0.374 | (0.058, 0.691) |  |  |
|  | Other Hispanic |  | 0.391 | (0.061, 0.720) |  |  |  | 0.381 | (-0.004, 0.766) |  |  |


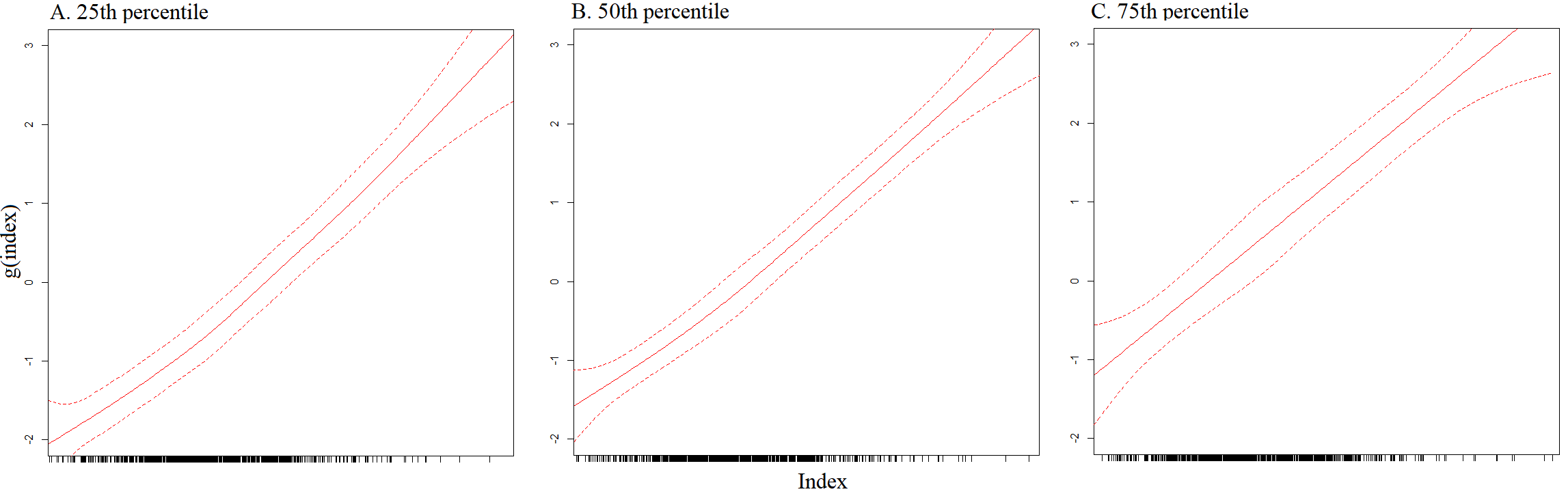


Figure S4. Estimated link functions by PLSI quantile regressions at three quartiles in NHANES 2002-2003. (a) 25th percentile; (b) 50th percentile; (c) 75th percentile.

Table S5. Results from PLSI logistic regression and multivariable logistic regression in NHANES 2002-2003.

| Variable | PLSI logistic rank | PLSI logistic estimate | PLSI logistic 95% CI | PLSI logistic Proportion of contribution (%) |  | Logistic rank | Logistic original estimate | Logistic original 95% CI | Logistic normed estimate | Logistic normed 95% CI |
| --- | --- | --- | --- | --- | --- | --- | --- | --- | --- | --- |
| Environmental factors |  |  |  |  |  |  |  |  |  |  |
| a-Tocopherol | 1 | 0.584 | (0.433, 0.735) | 34.1 |  | 1 | 1.290 | (0.899, 1.682) | 0.584 | (0.448, 0.720) |
| g-tocopherol | 4 | 0.381 | (0.273, 0.490) | 14.5 |  | 4 | 0.842 | (0.580, 1.104) | 0.381 | (0.279, 0.483) |
| Retinyl-palmitate | 2 | 0.460 | (0.294, 0.627) | 21.2 |  | 2 | 1.018 | (0.695, 1.340) | 0.460 | (0.313, 0.608) |
| Retinol | 8 | 0.107 | (-0.008, 0.223) | 1.1 |  | 8 | 0.237 | (-0.018, 0.492) | 0.107 | (-0.001, 0.215) |
| 3,3,4,4,5-pncb | 7 | 0.117 | (0.000, 0.233) | 1.4 |  | 7 | 0.257 | (-0.023, 0.538) | 0.117 | (0.005, 0.228) |
| PCB194 | 5 | -0.250 | (-0.438, -0.061) | 6.2 |  | 5 | -0.552 | (-0.938, -0.167) | -0.250 | (-0.419, -0.081) |
| 2.3.4.6.7.8.hxcdf | 6 | -0.235 | (-0.368, -0.102) | 5.5 |  | 6 | -0.519 | (-0.775, -0.263) | -0.235 | (-0.358, -0.112) |
| trans.b.carotene | 3 | -0.399 | (-0.520, -0.278) | 15.9 |  | 3 | -0.882 | (-1.157, -0.606) | -0.399 | (-0.507, -0.291) |
| Covariates |  |  |  |  |  |  |  |  |  |  |
| Intercept |  | -1.787 | (-3.038, -0.536) |  |  |  | -1.787 | (-2.970, -0.603) |  |  |
| Age |  | 0.014 | (-0.009, 0.036) |  |  |  | 0.014 | (-0.005, 0.033) |  |  |
| Sex (female) |  | -0.208 | (-0.699, 0.283) |  |  |  | -0.208 | (-0.697, 0.281) |  |  |
| Ethnicity |  |  |  |  |  |  |  |  |  |  |
| Non-Hispanic white |  | Ref |  |  |  |  | Ref |  |  |  |
| Non-Hispanic black |  | -0.198 | (-0.901, 0.505) |  |  |  | -0.198 | (-0.861, 0.465) |  |  |
| Mexican American |  | 0.696 | (0.085, 1.307) |  |  |  | 0.696 | (0.091, 1.301) |  |  |
| Other race |  | 1.851 | (0.781, 2.920) |  |  |  | 1.851 | (0.746, 2.955) |  |  |
| Other Hispanic |  | 0.696 | (-0.622, 2.014) |  |  |  | 0.696 | (-0.51, 1.902) |  |  |


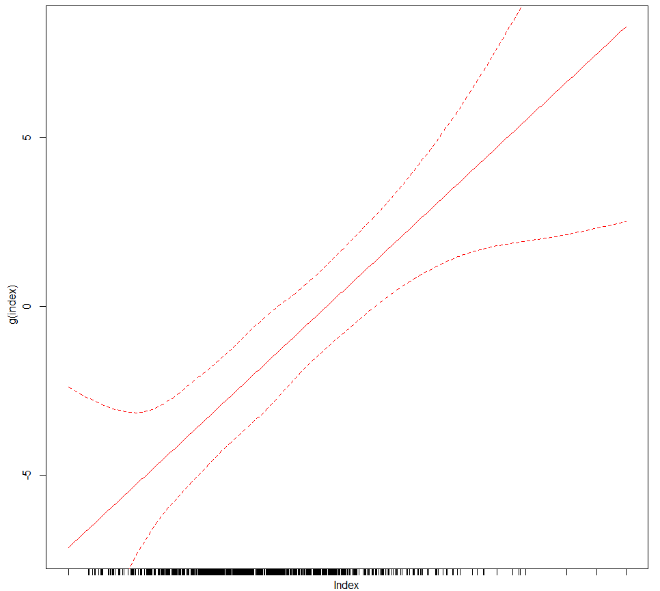


Figure S5. Estimated link function by PLSI logistic regression in NHANES 2002-2003.


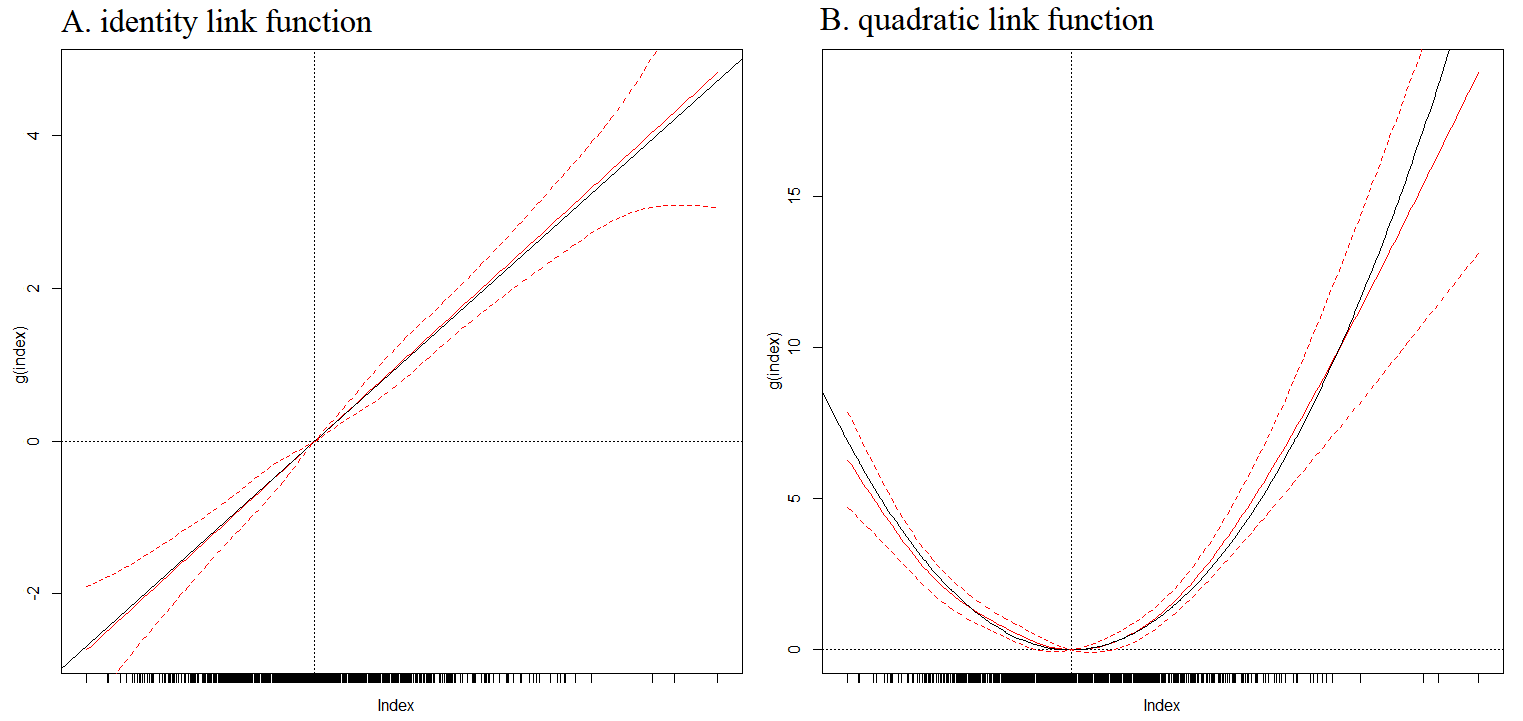


Figure S6. Estimated link functions by PLSI PH model in simulated time-to-event study. (a) identity link function; (b) quadratic link function. The black line was the underlying true link function, and the red solid line was the estimated link function with dotted lines of 95% point-wise confidence intervals.


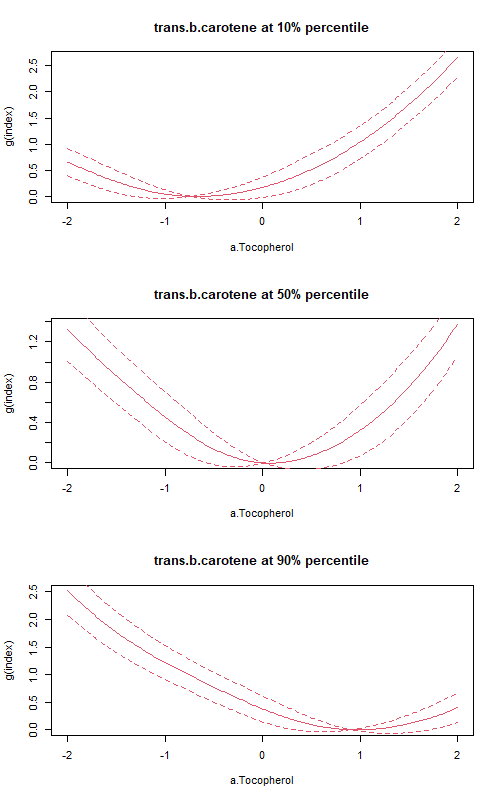


Figure S7. Stratified effect of a-Tocopherol with 95% confidence intervals when the variable of trans-b-.carotene fixed at 10%, 50%, and 90% percentile and other factors fixed as median values.

Table S6. Simulation results from PLSI PH model and Cox PH model for link function $g\left( x \right)=0.2x^{3}-x^{2}+3x$.

| Variable | True rank | True coefficient |  | PLSI PH rank | PLSI PH estimate | PLSI PH 95% CI | PLSI PH  Proportion of contribution (%) |  | Cox PH rank | Cox PH original estimate | Cox PH original 95% CI | Cox PH normed estimate | Cox PH normed 95% CI |
| --- | --- | --- | --- | --- | --- | --- | --- | --- | --- | --- | --- | --- | --- |
| Identity link function |  |  |  |  |  |  |  |  |  |  |  |  |  |
| Environmental factors |  |  |  |  |  |  |  |  |  |  |  |  |  |
| a-Tocopherol | 1 | 0.560 |  | 1 | 0.552 | (0.481, 0.623) | 30.5 |  | 1 | 1.050 | (0.854, 1.245) | 0.541 | (0.456, 0.625) |
| g-tocopherol | 2 | 0.490 |  | 2 | 0.500 | (0.426, 0.574) | 25.0 |  | 2 | 0.925 | (0.772, 1.077) | 0.476 | (0.403, 0.549) |
| Retinyl-palmitate | 3 | 0.420 |  | 3 | 0.446 | (0.355, 0.537) | 19.9 |  | 3 | 0.904 | (0.714, 1.095) | 0.466 | (0.362, 0.569) |
| Retinol | 7 | 0.140 |  | 7 | 0.115 | (0.029, 0.200) | 1.3 |  | 7 | 0.257 | (0.108, 0.405) | 0.132 | (0.048, 0.216) |
| 3,3,4,4,5-pncb | 8 | 0.070 |  | 8 | 0.063 | (-0.025, 0.151) | 0.4 |  | 8 | 0.113 | (-0.067, 0.294) | 0.058 | (-0.039, 0.156) |
| PCB194 | 6 | -0.210 |  | 6 | -0.161 | (-0.274, -0.047) | 2.6 |  | 6 | -0.329 | (-0.574, -0.084) | -0.170 | (-0.288, -0.051) |
| 2.3.4.6.7.8.hxcdf | 5 | -0.280 |  | 5 | -0.312 | (-0.396, -0.227) | 9.7 |  | 5 | -0.618 | (-0.769, -0.467) | -0.318 | (-0.403, -0.234) |
| trans.b.carotene | 4 | -0.350 |  | 4 | -0.326 | (-0.401, -0.251) | 10.6 |  | 4 | -0.653 | (-0.811, -0.495) | -0.336 | (-0.414, -0.259) |
| Covariates |  |  |  |  |  |  |  |  |  |  |  |  |  |
| Age |  | 0.005 |  |  | 0.009 | (-0.004, 0.021) |  |  |  | 0.009 | (-0.003, 0.022) |  |  |
| Sex (female) |  | -0.076 |  |  | -0.094 | (-0.448, 0.260) |  |  |  | -0.080 | (-0.381, 0.220) |  |  |
| Ethnicity |  |  |  |  |  |  |  |  |  |  |  |  |  |
| Non-Hispanic white |  | Ref |  |  | Ref |  |  |  |  | Ref |  |  |  |
| Non-Hispanic black |  | -0.138 |  |  | 0.052 | (-0.382, 0.486) |  |  |  | 0.159 | (-0.255, 0.573) |  |  |
| Mexican American |  | 0.175 |  |  | 0.271 | (-0.123, 0.665) |  |  |  | 0.350 | (-0.026, 0.725) |  |  |
| Other race |  | 0.409 |  |  | 1.750 | (1.044, 2.456) |  |  |  | 1.871 | (1.246, 2.496) |  |  |
| Other Hispanic |  | 0.355 |  |  | 0.474 | (-9.300, 10.248) |  |  |  | 0.481 | (-0.273, 1.235) |  |  |


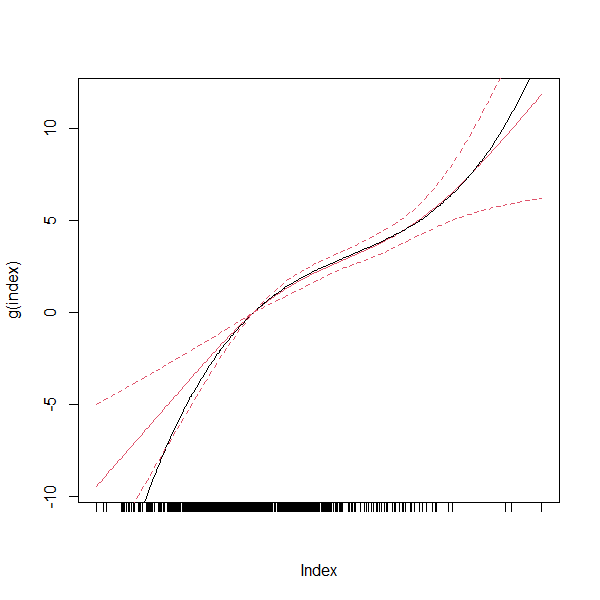


Figure S8. Estimated link functions by PLSI PH model in simulated time-to-event study with link function $g\left( x \right)=0.2x^{3}-x^{2}+3x$. The black line was the underlying true link function, and the red solid line was the estimated link function with dotted lines of 95% point-wise confidence intervals.


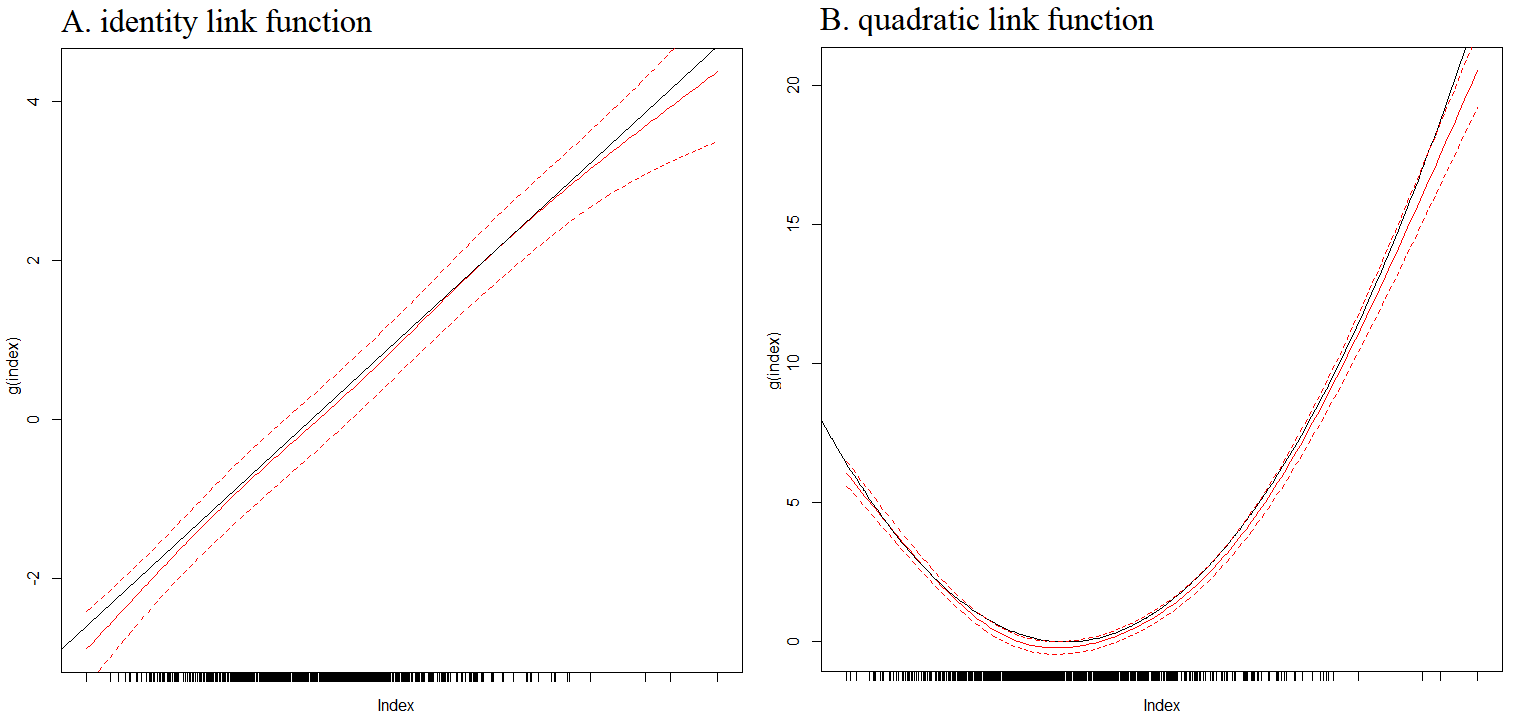


Figure S9. Estimated link functions by PLSI mixed-effects model in simulated longitudinal study. (a) identity link function; (b) quadratic link function. The black line was the underlying true link function, and the red solid line was the estimated link function with dotted lines of 95% point-wise confidence intervals.


Table S7. Sensitivity analysis results from weighted PLSI linear regression and weighted linear regression in NHANES 2002-2003 using NHANES laboratory subsample C weights.

| Variable | PLSI LR rank | PLSI  LR estimate | PLSI  LR 95% CI | PLSI LR Proportion of contribution (%) |  | LR rank | LR original estimate | LR original 95% CI | LR normed estimate | LR normed 95% CI |
| --- | --- | --- | --- | --- | --- | --- | --- | --- | --- | --- |
| Environmental factors |  |  |  |  |  |  |  |  |  |  |
| a-Tocopherol | 1 | 0.597 | (0.502, 0.692) | 35.6 |  | 1 | 0.456 | (0.391, 0.521) | 0.621 | (0.509, 0.734) |
| g-tocopherol | 2 | 0.421 | (0.347, 0.495) | 17.7 |  | 2 | 0.297 | (0.255, 0.339) | 0.405 | (0.321, 0.489) |
| Retinyl-palmitate | 4 | 0.395 | (0.298, 0.493) | 15.6 |  | 3 | 0.293 | (0.236, 0.351) | 0.400 | (0.284, 0.516) |
| Retinol | 7 | 0.162 | (0.088, 0.236) | 2.6 |  | 8 | 0.116 | (0.066, 0.167) | 0.158 | (0.070, 0.246) |
| 3,3,4,4,5-pncb | 8 | 0.146 | (0.071, 0.221) | 2.1 |  | 7 | 0.118 | (0.063, 0.173) | 0.161 | (0.076, 0.246) |
| PCB194 | 6 | -0.223 | (-0.342, -0.103) | 5.0 |  | 6 | -0.128 | (-0.208, -0.048) | -0.174 | (-0.300, -0.048) |
| 2.3.4.6.7.8.hxcdf | 5 | -0.227 | (-0.306, -0.147) | 5.1 |  | 5 | -0.166 | (-0.214, -0.119) | -0.227 | (-0.320, -0.133) |
| trans.b.carotene | 3 | -0.402 | (-0.475, -0.329) | 16.2 |  | 4 | -0.291 | (-0.338, -0.244) | -0.397 | (-0.481, -0.312) |
| Covariates |  |  |  |  |  |  |  |  |  |  |
| Intercept |  | 0.052 | (-0.195, 0.299) |  |  |  | 0.138 | (-0.077, 0.352) |  |  |
| Age |  | 0.002 | (-0.002, 0.007) |  |  |  | 0.000 | (-0.004, 0.004) |  |  |
| Sex (female) |  | -0.121 | (-0.213, -0.029) |  |  |  | -0.122 | (-0.215, -0.03) |  |  |
| Ethnicity |  |  |  |  |  |  |  |  |  |  |
| Non-Hispanic white |  | Ref |  |  |  |  | Ref |  |  |  |
| Non-Hispanic black |  | -0.091 | (-0.217, 0.036) |  |  |  | -0.072 | (-0.216, 0.073) |  |  |
| Mexican American |  | 0.169 | (0.048, 0.291) |  |  |  | 0.190 | (0.020, 0.360) |  |  |
| Other race |  | 0.347 | (0.080, 0.614) |  |  |  | 0.316 | (0.106, 0.526) |  |  |
| Other Hispanic |  | 0.233 | (-0.039, 0.505) |  |  |  | 0.233 | (0.000, 0.466) |  |  |
